# Genome sequence of the progenitor of wheat A subgenome *Triticum urartu*

**DOI:** 10.1038/s41586-018-0108-0

**Published:** 2018-05-09

**Authors:** Hong-Qing Ling, Bin Ma, Xiaoli Shi, Hui Liu, Lingli Dong, Hua Sun, Yinghao Cao, Qiang Gao, Shusong Zheng, Ye Li, Ying Yu, Huilong Du, Ming Qi, Yan Li, Hongwei Lu, Hua Yu, Yan Cui, Ning Wang, Chunlin Chen, Huilan Wu, Yan Zhao, Juncheng Zhang, Yiwen Li, Wenjuan Zhou, Bairu Zhang, Weijuan Hu, Michiel J. T. van Eijk, Jifeng Tang, Hanneke M. A. Witsenboer, Shancen Zhao, Zhensheng Li, Aimin Zhang, Daowen Wang, Chengzhi Liang

**Affiliations:** 1 State Key Laboratory of Plant Cell and Chromosome Engineering, Institute of Genetics and Developmental Biology, Chinese Academy of Sciences, Beijing, China; 2 College of Life Sciences, University of Chinese Academy of Sciences, Beijing, China; 3 State Key Laboratory of Plant Genomics, Institute of Genetics and Developmental Biology, Chinese Academy of Sciences, Beijing, China; 4 Keygene N.V., Wageningen, The Netherlands; 5 BGI-Shenzhen, Shenzhen, China

**Keywords:** DNA sequencing, Genome duplication

## Abstract

*Triticum urartu* (diploid, AA) is the progenitor of the A subgenome of tetraploid (*Triticum turgidum*, AABB) and hexaploid (*Triticum aestivum*, AABBDD) wheat^1,2^. Genomic studies of *T. urartu* have been useful for investigating the structure, function and evolution of polyploid wheat genomes. Here we report the generation of a high-quality genome sequence of *T. urartu* by combining bacterial artificial chromosome (BAC)-by-BAC sequencing, single molecule real-time whole-genome shotgun sequencing^3^, linked reads and optical mapping^4,5^. We assembled seven chromosome-scale pseudomolecules and identified protein-coding genes, and we suggest a model for the evolution of *T. urartu* chromosomes. Comparative analyses with genomes of other grasses showed gene loss and amplification in the numbers of transposable elements in the *T. urartu* genome. Population genomics analysis of 147 *T. urartu* accessions from across the Fertile Crescent showed clustering of three groups, with differences in altitude and biostress, such as powdery mildew disease. The *T. urartu* genome assembly provides a valuable resource for studying genetic variation in wheat and related grasses, and promises to facilitate the discovery of genes that could be useful for wheat improvement.

## Abstract (web summary)

The genome sequence of *Triticum urartu*, the progenitor of the A subgenome of hexaploid wheat, provides insight into genome duplication during grass evolution.

## Main

**Extended Data Fig. 1:**
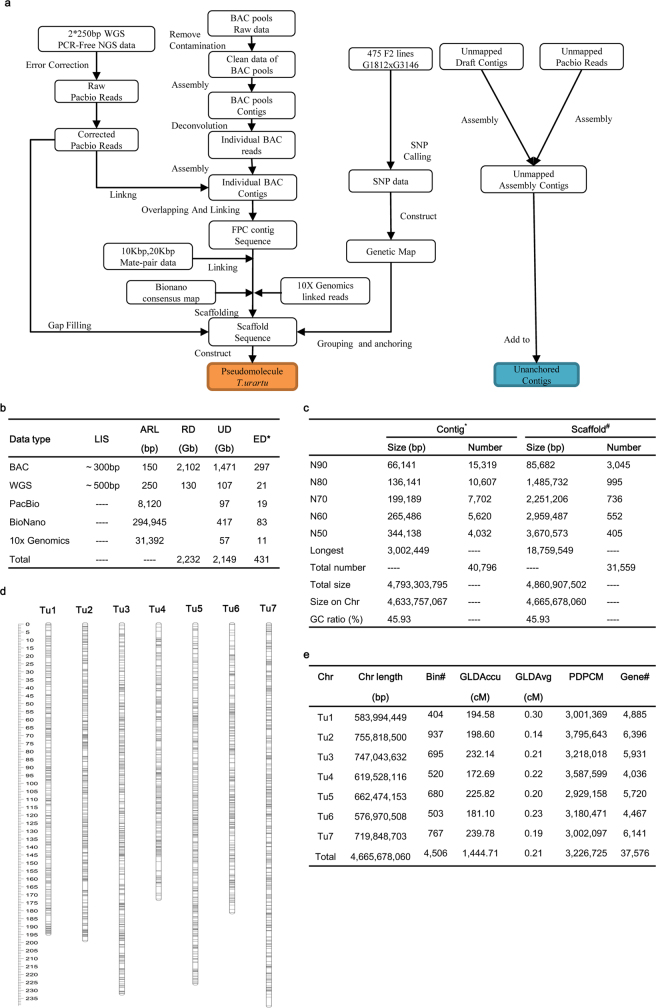
*T. urartu* genome assembly. **a**, Schematic workflow for genome sequencing, assembly and chromosomal assignment with a high-density SNP map. **b**, Statistics of sequencing data. *Calculated from the estimated genome size of 4.94 Gb. LIS, library insert size; ARL, average read length; RD, raw data; UD, usable data; ED, effective depth. **c**, Summary of the Tu genome assembly. *Contig: contiguous sequence without Ns assembled with Illumina reads, corrected by PacBio reads. ^#^Scaffold: Sequence with Ns, in which two or more contigs were connected by mate-pair reads, BioNano genome maps and 10× Genomics linked reads. **d**, High-resolution genetic map of *T. urartu* using SNP markers. The SNP markers were identified from an F2 population (475 individuals) derived from a cross between accessions G1812 and G3146 of Tu. **e**, Summary of physical length and genetic map of seven pseudomolecules. Chr, chromosome; GLDAccu, genetic linkage distance accumulation; GLDAvg, genetic linkage distance on average; PDPCM, physical distance per centiMorgen (cM).

**Extended Data Fig. 2:**
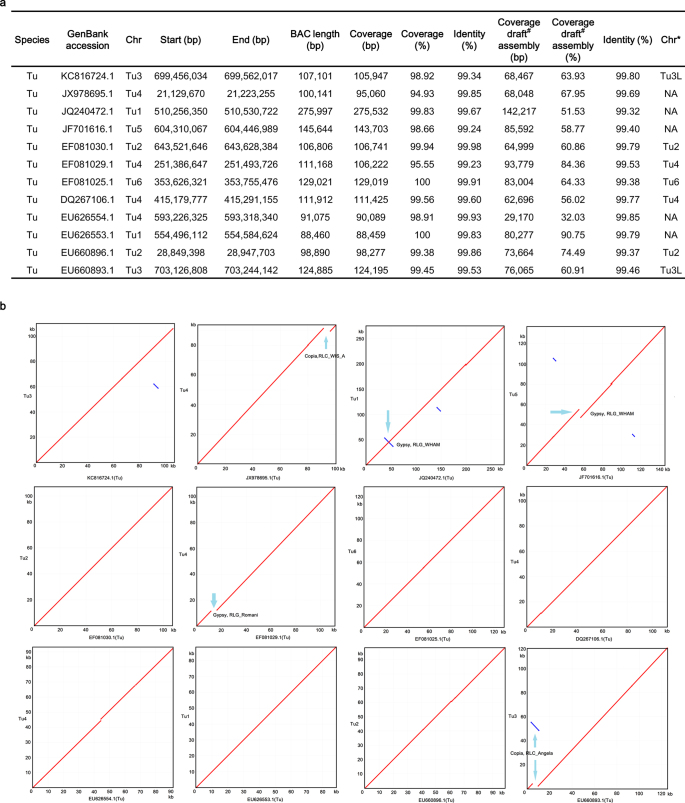
Evaluation of *T. urartu* genome assembly. **a**, Summary of comparison of the *T. urartu* genome assembly with public BAC sequences. *The published chromosome location of BACs; NA, not available. ^#^Tu draft assembly^6^. **b**, Dot plots showing comparison of *T. urartu* genome with available BACs of *T. urartu* from public database. The blue arrows indicate the regions at which the BAC sequences and pseudomolecules did not match owing to the presence of repeat elements.

The genome of *T. urartu* (Tu) accession G1812 (PI428198) was sequenced and assembled (Extended Data Fig. 1a–c). The assembled contig sequences were 4.79 Gb with an N50 (the length *N* for which 50% of all bases in the sequences are in a sequence of length *L* < N) of 344 kb and scaffold sequences were 4.86 Gb with an N50 of 3.67 Mb (Table 1), very close to the estimated genome size of 4.94 Gb^6^. We anchored 4.67 Gb (95.9%) of the scaffold sequences onto Tu chromosomes with a high density single nucleotide polymorphism (SNP) genetic map (Extended Data Fig. 1d), generating seven DNA pseudomolecules (Supplementary Data 1, Extended Data Fig. 1e). The high quality of the assembled sequences was confirmed at both the nucleotide and chromosome levels by comparison with previously published BAC sequences and the draft genome sequence of *Triticum aestivum* (Ta)^7^ (Extended Data Fig. 2, Supplementary Data 2).

**Table 1:** Summary of the Tu genome assembly and annotation

**Genome assembly**	Estimated genome size	4.94 Gb	
	GC content	45.93%	
	N50 length (contig)	344 kb	
	Longest contig	3.00 Mb	
	Total length of contigs	4.79 Gb	
	N50 length (scaffold)	3.67 Mb	
	Longest scaffold	18.76 Mb	
	Total length of scaffolds	4.86 Gb	
**Transposable elements**	**Annotation**	**Per cent**	**Total length**
	Retrotransposons	71.83	3.44 Gb
	DNA transposons	7.41	0.35 Gb
	Others	2.19	0.10 Gb
	Total	81.42	3.90 Gb
**Protein-coding genes**	Predicted genes	41,507	
	Average transcript length	1,453 bp	
	Average coding sequence length	998 bp	
	Average exon length	320 bp	
	Average intron length	508 bp	
	Functionally annotated	36,602	

We predicted 41,507 protein-coding genes, including 37,516 high-confidence and 3,991 low-confidence genes (Extended Data Table 1a) using the Gramene pipeline^8^. On average, the genes have transcript length of 1,453 bp, protein length of 332 amino acids, and 4.5 exons per transcript, which were comparable to genes in other grasses^9,10^ (Extended Data Table 1b). Approximately 88.18% of the predicted genes were assigned functional annotations (Extended Data Table 1c). We also predicted that 10,514 genes could produce alternatively spliced transcripts, with an average of 2.95 transcripts per gene. Moreover, we identified 31,269 microRNAs (miRNAs), 5,810 long non-coding RNAs (lncRNAs), 3,620 transfer RNAs (tRNAs), 80 ribosomal RNAs (rRNAs) and 2,519 small nuclear RNAs (snRNAs) throughout the genome (Extended Data Table 1d).

**Fig. 1:**
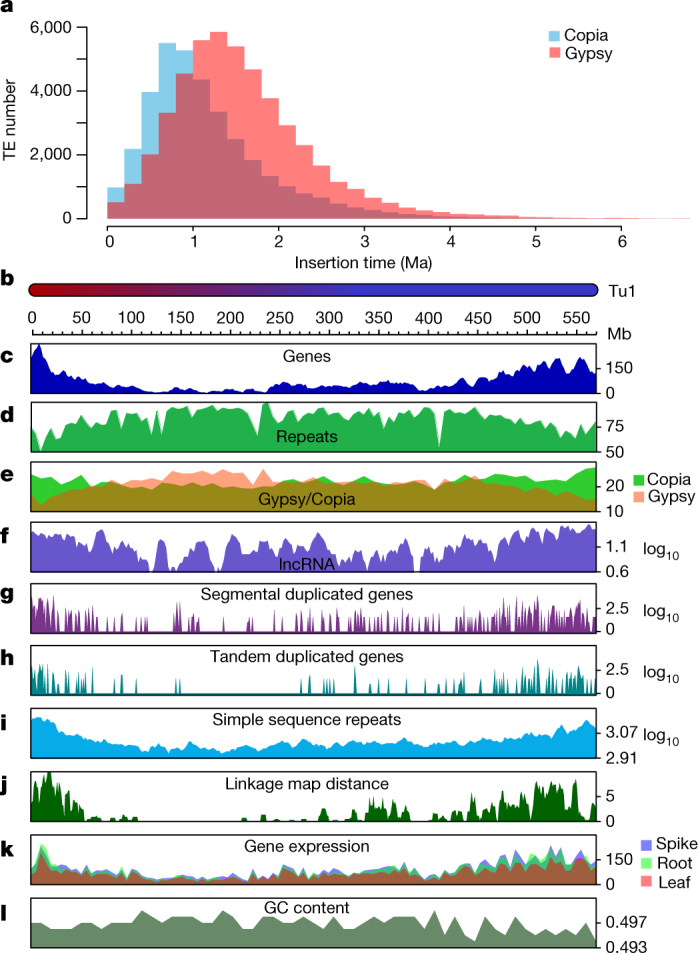
Recent LTR retrotransposon bursts in the Tu genome and distribution of genomic components on Tu chromosome 1. **a**, Insertion burst of LTR retrotransposons of Gypsy and Copia. TE, transposable element. **b**–**l**, Multi-dimensional display of genomic components of Tu chromosome 1. **b**, DNA pseudomolecule. **c**, Gene frequency (number of genes per 10 Mb). **d**, Repeat density (per cent nucleotides per 5 Mb). **e**, Density of LTR retrotransposons (per cent nucleotides per 10 Mb). **f**, Frequency of lncRNA (log[number per 10 Mb]). **g**, Frequency of segmentally duplicated genes (log[number per 1 Mb]). **h**, Frequency of tandemly duplicated genes (log[number per 1 Mb]). **i**, Frequency of simple sequence repeats (log[number per 10 Mb]). **j**, Linkage map distance (cM per 5 Mb). **k**, Accumulated gene expression level (log_2_[FPKM (fragments per kilobase of transcript per million mapped reads) per 5 Mb]). **l**, GC content (per cent per 1 Mb).

A total of 3.90 Gb (81.42%) of genome sequences was identified as repetitive elements, including 3.44 Gb (71.83%) of retrotransposons and 355 Mb (7.41%) of DNA transposons (Extended Data Table 1e). Among long-terminal repeat (LTR) retrotransposons, the Gypsy and Copia superfamilies comprised 42.71% and 24.30% of the genome, respectively. Further, we identified 48,370 intact Gypsy retrotransposons for which the peak of amplification bursts appeared at more than one million years ago (Ma) and 35,559 intact Copia retrotransposons with a peak less than 1 Ma (Fig. 1a). We also identified 121,792 solo-LTR/Gypsy and 44,349 solo-LTR/Copia elements, yielding solo-LTR/intact element ratios of 2.5 and 1.2 for Gypsy and Copia retrotransposons, respectively. These results showed an earlier burst of Gypsy retrotransposition than of Copia retrotransposition in the Tu genome; both bursts occurred after the divergence of the A and B genomes^11^.

**Extended Data Fig. 3:**
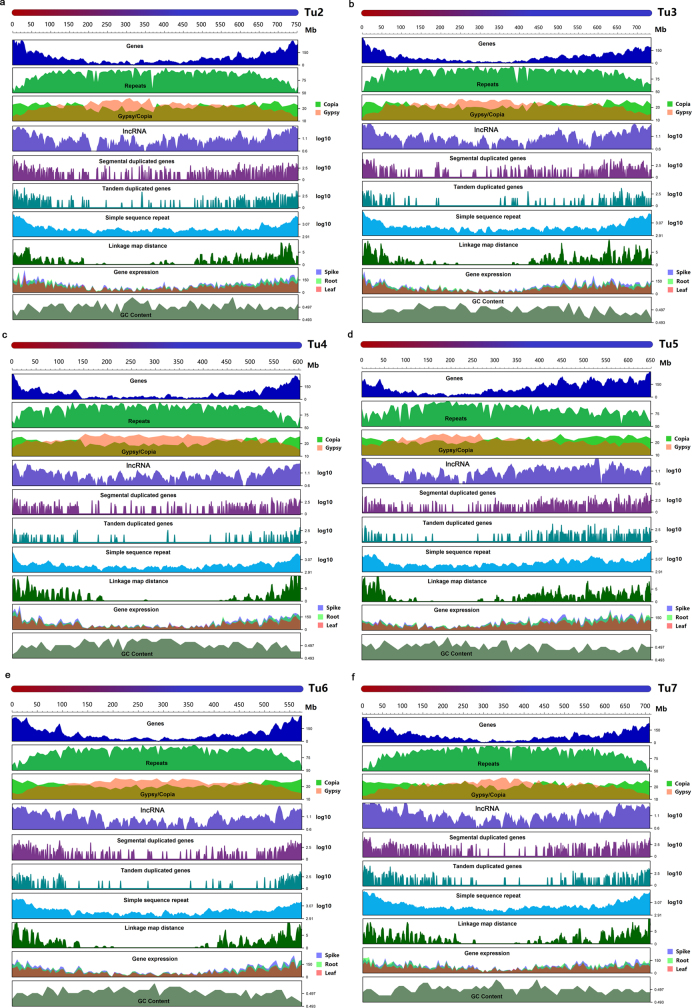
Chromosomal distribution of *T. urartu* genome features. **a**–**f**, Features on Tu2–Tu7 are in the order of DNA pseudomolecule; gene frequency (number of genes per 10 Mb); repeat density (per cent nucleotides per 5 Mb); density of LTR retrotransposons (per cent nucleotides per 10 Mb); frequency of lncRNA (log[number of genes per 10 Mb]); frequency of segmentally duplicated genes (log[number of genes per 1 Mb]); frequency of tandemly duplicated genes (log[number of genes per 1 Mb]); frequency of simple sequence repeats (log[number of repeats per 10 Mb]); linkage map distance (cM per 5 Mb); accumulated gene expression level in RNA-seq data (log_2_[FPKM per 5 Mb]); GC content (per cent per 1 Mb).

We found substantially higher gene density and recombination rates, as well as lower densities of transposable elements and tandem repeats, near the telomeres of each chromosome (Fig. 1b–l, Extended Data Fig. 3). LTR retrotransposons were distributed unevenly throughout each chromosome. The distribution of Copia elements was enriched at both telomeric–subtelomeric regions, whereas Gypsy retrotransposons were enriched in the pericentromeric–centromeric regions (Fig. 1e, Extended Data Fig. 3). The accumulated gene expression level was higher in subtelomeres than in the centromeric regions (Fig. 1k, Extended Data Fig. 3).

**Extended Data Fig. 4:**
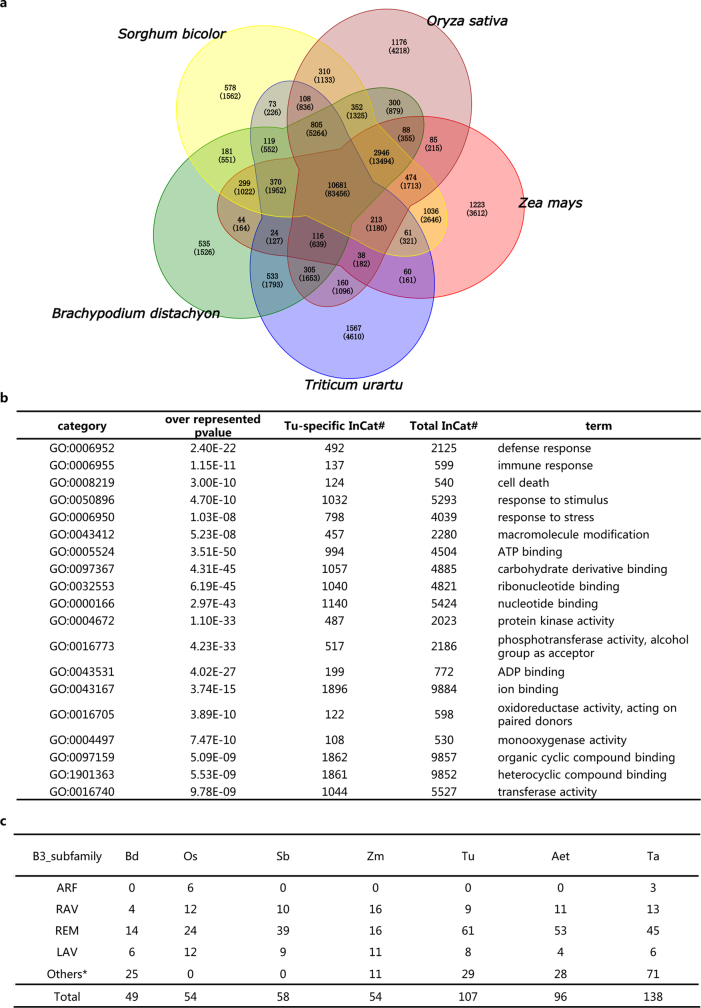
Analyses of gene families and B3 transcription factors. **a**, Comparison of gene families of *T. urartu* with *O. sativa*, *Z. mays*, *S. bicolor* and *B. distachyon*. Venn diagram illustrates shared and unique gene families (gene numbers in parentheses) among the five grass species. **b**, Gene ontology analysis of Tu-specific genes. Tu-specific InCat#, number of Tu-specific genes in the GO category; Total InCat#, number of total Tu genes in the GO category. **c**, Comparison of B3 transcription factors of Tu with *B. distachyon* (Bd), *O. sativa* (Os), *S. bicolor* (Sb), *Z. mays* (Zm), *Ae. tauschii* (Aet) and *T. aestivum* (Ta). *B3 transcription factors without identified subfamily.

Analyses of genes in the Tu genome together with those from rice^9^, maize^12^, sorghum^13^ and *Brachypodium*^10^ were clustered into 24,860 gene families (Extended Data Fig. 4a). Of these, 10,681 families were shared among the five examined plant genomes, representing a core set of genes across these grass genomes. There were 4,610 genes from 1,567 gene families that were specific to Tu, of which many have functional gene ontology annotations relating to responses to stimulus and stress (Extended Data Fig. 4b).

By comparing transcription factors in the Tu genome with those of the six sequenced grass genomes in the iTAK^14^ collection, including *Brachypodium*, rice, sorghum, maize, *Aegilops tauschii* (Aet)^15^ and Ta^7,16^, we found that the number of reproductive meristem (REM) subfamily genes in the transcription factor B3 family^17^ was amplified in the genomes of Tu, Aet and Ta (Supplementary Data 3, Extended Data Fig. 4c, Supplementary Information S1.1). The REM subfamily is functionally related to vernalization and flower development^18^. Therefore, we speculate that the amplification of B3 REM transcription factors in wheat genomes might be related to the vernalization process of wheat. Furthermore, we identified 598 disease resistance genes (Supplementary Data 4) and 22 prolamin genes (Supplementary Data 5, Supplementary Information S1.2).

**Fig. 2:**
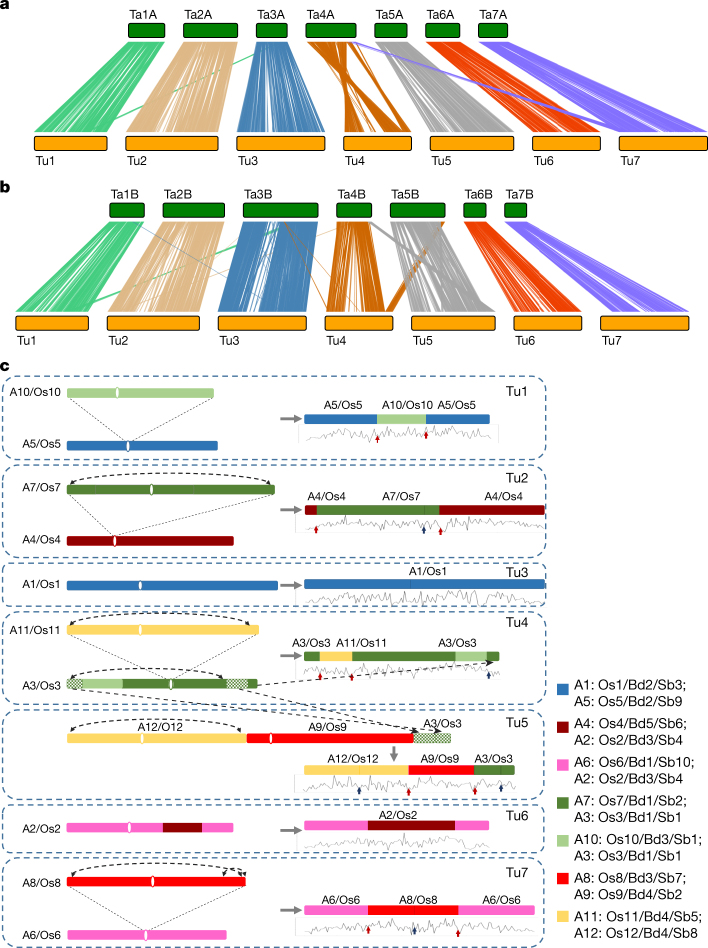
Genome synteny to bread wheat (Ta) and an evolutionary model of the Tu chromosomes. **a**, **b**, Synteny of Tu chromosomes with subgenomes A and B of Ta. Each line represents a syntenic block of five or more gene pairs with similarity of 80% or more. Three large structural variations detected are: (1) a reciprocal translocation at the distal end of the long arms between Tu4 and Tu5 that occurred before the polyploidization of the A and B genomes, but after divergence from both B and D genomes; (2) a one-way translocation from Ta7B to Ta4A; and (3) a pericentric inversion on Ta4A involving most of the long and short arms. **c**, Evolutionary model of Tu chromosomes from an ancestral grass genome based on the AGK structure initially defined in Murat et al.^24^ and the syntenic relationships of Tu with *B. distachyon* (Bd), rice (Os), and sorghum (Sb). One-directional arrows indicate segment translocations, and bidirectional arrows indicate inversions. Tu1–Tu7, seven chromosomes of Tu; A1–A12, twelve chromosomes of the grass ancestor; Bd1–Bd5, five chromosomes of Bd; Os1–Os12, twelve rice chromosomes; Sb1–Sb10, ten sorghum chromosomes. The seven coloured squares on the right represent seven basic ancient grass chromosomes^24^. The line graphs below Tu chromosomes display the frequency distribution of AGK genes. The red and blue arrows indicate inter- and intra-chromosome fusion locations, respectively, of the ancestral chromosomes in the Tu genome.

**Extended Data Fig. 5:**
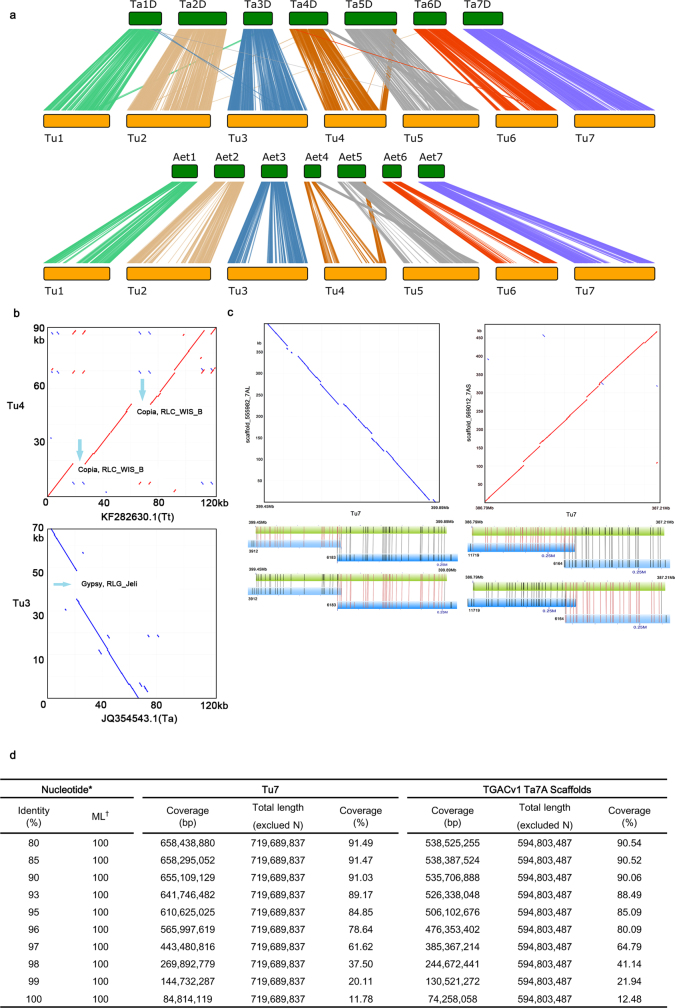
Comparison of Tu genome with other wheat genomes. **a**, Syntenic analysis of Tu genome with the D subgenome of Ta, and the genome of Aet. Each syntenic block contains five or more genes, with sequence similarity of 80% or more. **b**, Comparison of Tu genome with BACs of *T. turgidum* (Tt) and Ta. BAC KF282630 from chromosome 4 of the A subgenome of Tt contained two inserted fragments (blue arrows) that are composed of Copia RLC_WIS_B elements, which were not detected on the corresponding Tu4 region. BAC JQ354543 from chromosome 3 of the A subgenome of Ta lacked the Gypsy RLG_Jeli element (blue arrow), which was found on the corresponding region of Tu3. **c**, Comparison of the Tu genome with Ta7A scaffolds from TGACv1^19^. The dot plots on the top show comparison of two largest TaA scaffolds to corresponding parts of Tu7 chromosome. The diagonal lines on the dot plots show fine co-linearity. The lower part shows validation of the sequence assembly of Tu7 by BioNano maps. The Tu7 sequences were digested into in silico consensus maps, and the consensus maps corresponding to the two Ta7A scaffolds (green bar) are compared against their corresponding BioNano genome maps (blue bar). Each vertical line on the green/blue bars indicates a restriction enzyme cutting site (Nt.BspQI), and vertical lines between green bars and blue bars indicate alignments among these sites. The blue bars highlighted with red vertical line demonstrate that two BioNano genome maps (for example, 3912 and 6183) overlap with one another (they all have alignments on the overlapping region), although the two maps are not merged together owing to lack of coverage on the overlapping region. The high consistency of alignments between consensus maps and BioNano genome maps confirm the high quality of Tu genome assembly. Therefore, the insertion/deletion events in the dot plots should be sequence variations between the two A genomes from Ta and Tu, rather than assembly errors. **d**, Comparison of Tu7 with all Ta7A scaffolds from TGACv1 at nucleotide levels. *Nucleotide: minimum cutoff of DNA sequence alignments between Tu7 and Ta7A. ^†^ML, minimum length (bp) to align.

We identified three large structural variations that occurred in either Tu or Ta, with clearly defined boundaries, by comparing the Tu genome to the draft sequences of three sub-genomes of hexaploid wheat^7^ (Fig. 2a, b, Extended Data Fig. 5a, Supplementary Information S2.1). We aligned the Tu genome with sequences from six BACs of the A subgenome of *T. turgidum* (Tt) and eleven BACs from the A subgenome of Ta, and found that the unaligned regions between the BAC and the Tu genomic sequences resulted from the insertion of LTR retrotransposons in either Tu or Tt and/or Ta (Extended Data Fig. 5b). Furthermore, we compared the chromosome 7 assembly of the A subgenome of Ta^19^ (Ta7A) to Tu chromosome 7 (Tu7), and found that 655 Mb (91.03%) and 536 Mb (90.06%) of Tu7 and Ta7A sequences, respectively, were aligned to each other at a minimum identity of 90% or lower, with many unaligned retrotransposon regions (Extended Data Fig. 5c, d). These results show that the different wheat A genomes experienced large-scale structural rearrangements both before and after polyploidization with other genomes, and have experienced independent gain or loss of LTR retrotransposons after the polyploidization event.

**Extended Data Fig. 6:**
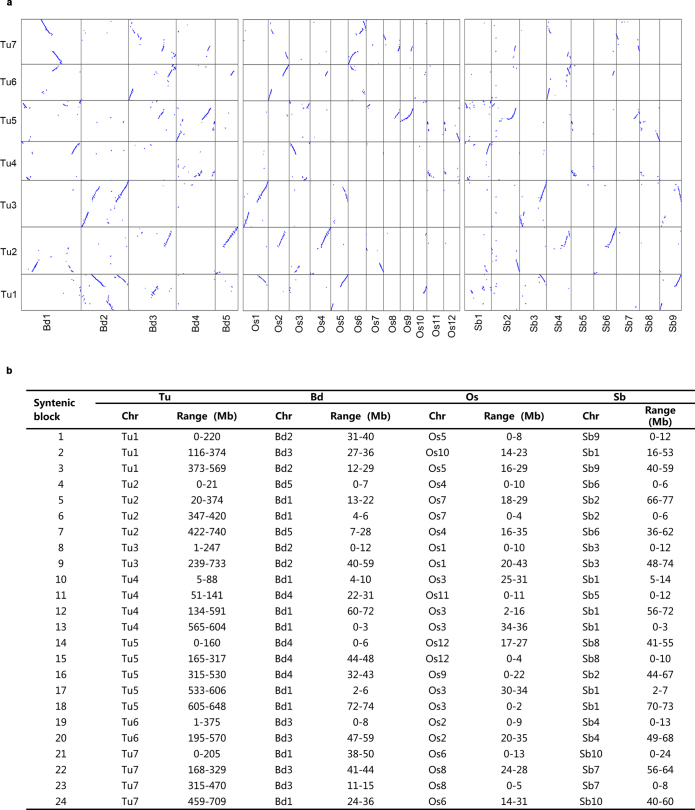
Synteny of *T. urartu* genome with other grass genomes. **a**, Intergenomic dot plots showing orthologous syntenies between Tu and three grass relatives *B. distachyon* (Bd), *O. sativa* (Os) and *S. bicolor* (Sb). The alignment of the Tu genome to the Bd, Os and Sb genomes demonstrates their highly collinear relationships and full coverage of Tu by orthologous chromosome segments from each of the three grass genomes. **b**, Data table showing the orthologous syntenies between Tu and the other genomes (Bd, Os and Sb).

Tu shared with rice, *Brachypodium* and sorghum a common grass ancestor^20^ with seven pairs of ancient chromosomes, which became 12 pairs of ancestral chromosomes (still maintained in rice) after one round of whole-genome duplication (WGD, 70 Ma) and two additional chromosomal fusions^21–24^. By studying the collinear relationships among these species (Extended Data Fig. 6, Supplementary Data 6), we found that Tu3 and Tu6 are the most conserved Tu chromosomes, each being derived from a single ancient chromosome shared by Os1, Bd2 and Sb3 and Os2, Bd3 and Sb4, respectively (where Os is *Oryza sativa*, Bd is *Brachypodium distachyon* and Sb is *Sorghum bicolor*). Chromosomes Tu1, Tu2, Tu4 and Tu7 are each composed of chromosomal segments originating from two different ancient chromosomes. Tu5, however, was derived from three ancient chromosomes (Fig. 2c, Supplementary Information S2.2). These results are consistent with the model proposed by Murat et al.^24^, and we further narrowed the fusion boundaries to small regions of approximately hundreds of kilobases.

Using an approach based on that described by Murat et al.^25^, we inferred 11,718 Tu ancestral grass karyotype (AGK) genes, which account for 31.2% of all chromosome localized Tu genes, slightly lower than the percentage detected in rice (32.4%) and considerably lower than that in *Brachypodium* (47.4%). The AGK genes were depleted in pericentromeric and subtelomeric regions, as well as chromosomal fusion locations (Fig. 2c, Supplementary Information S2.2).

**Extended Data Fig. 7:**
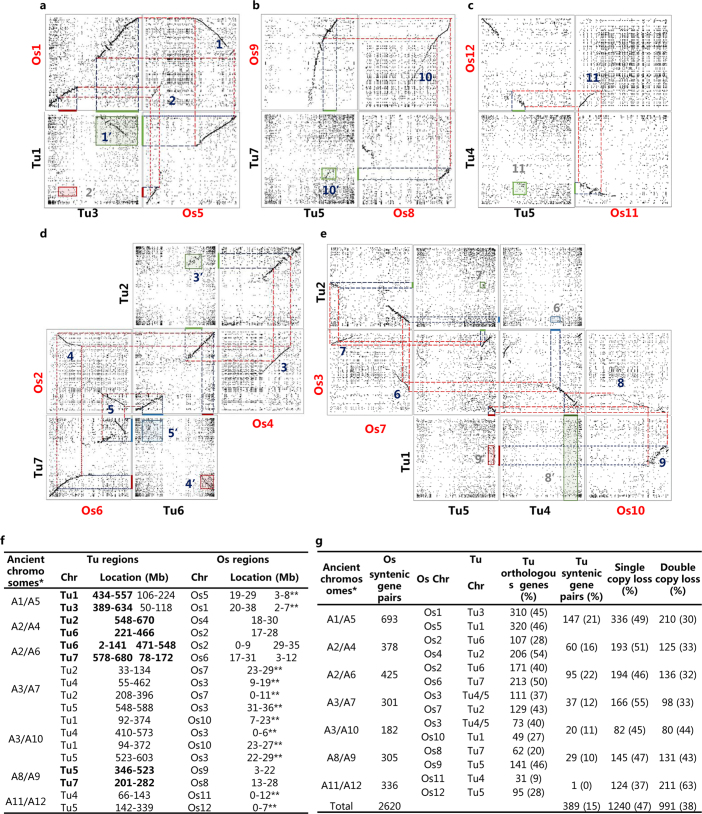
Intragenomic collinear regions of Tu corresponding to rice (Os) duplications. **a**, Intragenomic and intergenomic dot plots show a clearly visible collinear region 1′ between Tu1 and Tu3 that is orthologous to the Os intragenomic collinear region 1. However, the collinearity of region 2′ between Tu1 and Tu3 is severely corrupted, but the corresponding Os intragenomic collinear region 2 was clearly visible. **b**–**e**, Similarly, Tu intragenomic collinear regions 10′, 3′, 4′ and 5′ were also clearly visible, which correspond to Os intragenomic collinear regions 10, 3, 4 and 5, respectively. However, the collinearity of Tu intragenomic regions corresponding to Os intragenomic collinear regions 11, 6, 7, 8 and 9 was disrupted. **f**, Data table showing the strong intragenomic collinear segments of Os and their corresponding regions of Tu (chromosomal positions are shown in Mb). *The corresponding seven pairs of ancient chromosomes based on the AGK structure^24^ that are ancestral to the Tu and Os chromosomes listed in the right columns. The five clearly visible regions in Tu are marked in bold. **Os regions without visible corresponding collinear regions in Tu dot plots. **g**, Data table showing the number of collinear genes between ancestrally duplicated chromosome segments in rice and their corresponding gene numbers in Tu.

We performed intragenomic comparisons in Tu and found five clearly visible collinear regions in the dot plots (Extended Data Fig. 7, Supplementary Information S2.3). These regions originated from four pairs of anciently duplicated chromosomes^21,22^. However, compared to the rice genome, the collinearity was disrupted between each pair of the Tu chromosome segments derived from the remaining ancient chromosomes, owing to the loss of one or both copies of ancestral genes. For example, of the 2,620 anciently duplicated gene pairs still maintained in rice, approximately 47% and 38% had lost one and both copies, respectively, in the syntenic regions of Tu (Extended Data Fig. 7g).

**Extended Data Fig. 8:**
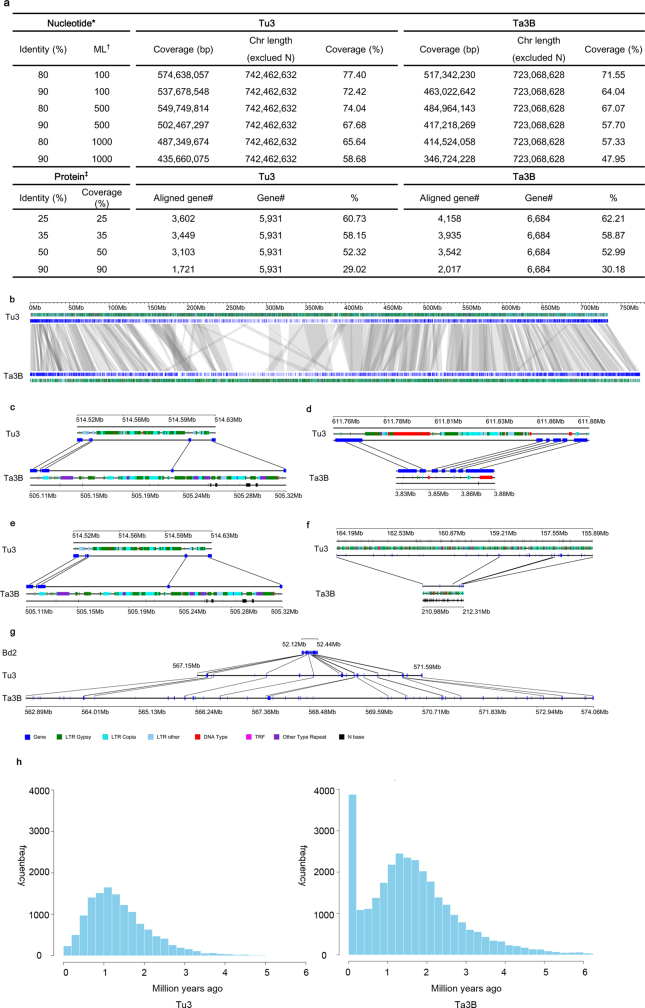
Comparison of chromosome 3 of Tu with chromosome 3B of Ta. **a**, Comparison of Tu3 with Ta3B at both nucleotide and protein levels. *Nucleotide: minimum cutoff of DNA sequence alignments between Tu3 and Ta3B. ^†^ML, minimum length (bp) to align. ^‡^Protein: minimum cutoff of protein sequence alignments between Tu3 and Ta3B, and in reverse. **b**, Overall view of syntenic blocks between Tu3 and Ta3B. **c**, A syntenic block composed of five consecutive collinear gene pairs, showing large repeat insertions on Ta3B of >100 kb. **d**, A syntenic block composed of seven consecutive collinear gene pairs. A 70-kb contraction is seen in Ta3B. **e**, A syntenic block with eight collinear genes interrupted by non-syntenic genes. **f**, Two segments of Tu3 and Ta3B show five discrete collinear gene pairs and gene expansions in Tu3. **g**, Genome expansion in one representative syntenic block from Bd2 (0.3 Mb), Tu3 (4.4 Mb), and Ta3B (11.2 Mb). Compared with the Tu3 segment, large numbers of non-homologous genes and repeats can be observed in Ta3B, resulting in a 7-Mb expansion. **h**, Insertion dates of LTR retrotransposons on Tu3 and Ta3B. A recent retrotransposon burst at around 0.1 Ma is observed on Ta3B but not on Tu3.

Comparative analysis of chromosomes Tu3 and Ta3B^26^ identified variations between the two chromosomes at both nucleotide and protein levels (Extended Data Fig. 8a–g, Supplementary Information S2.4). We identified 617 Mb (82.6%) and 651 Mb (84.1%) of syntenic sequences in Tu3 and Ta3B, respectively, with only 3,103 (52.32%) genes of Tu3 being aligned to 3,542 (52.99%) genes of Ta3B at a minimum protein identity of 50% and a minimum coverage of 50%. By comparison with syntenic genes from *Brachypodium*, rice and sorghum, we identified 393 and 213 deleted genes and 354 and 648 inserted genes within the collinear segments of Tu3 and Ta3B, respectively (Supplementary Data 7), highlighting the increased number of genes in Ta3B relative to Tu3. We also found a recent LTR retrotransposon burst in Ta3B, which was not observed in Tu3 (Extended Data Fig. 8h). Together, these differences may contribute to the larger size of Ta3B in comparison to Tu3, and suggest that amplification of LTR retrotransposons after the divergence of the A and B genomes may be relevant for wheat genome evolution.

**Fig. 3:**
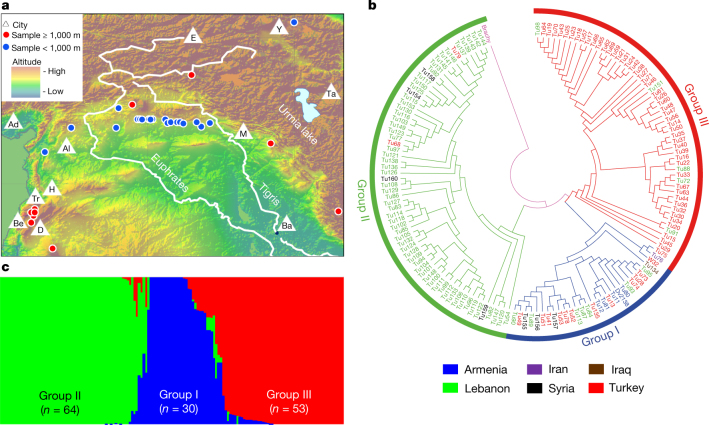
Geographic distribution and population structure of Tu. **a**, Distribution of the 147 Tu accessions at different altitudes of the Fertile Crescent. Also shown are the main land markers, including the Euphrates and Tigris rivers, Urmia lake and 11 cities (Adana (Ad), Aleppo (Al), Baghdad (Ba), Beirut (Be), Damascus (D), Erzurum (E), Homs (H), Mosul (M), Tabriz (Ta), Tripoli (Tr) and Yerevan (Y)). The map was drawn using the online mapping tool ArcGIS (version 10.1, www.esri.com). **b**, Phylogenetic clustering of the 147 accessions into three groups, with *B. distachyon* as the outgroup. **c**, Population structure analysis of Tu accessions, clustering with three groups that are similar to those from the phylogenetic analysis shown in **b**.

**Extended Data Fig. 9:**
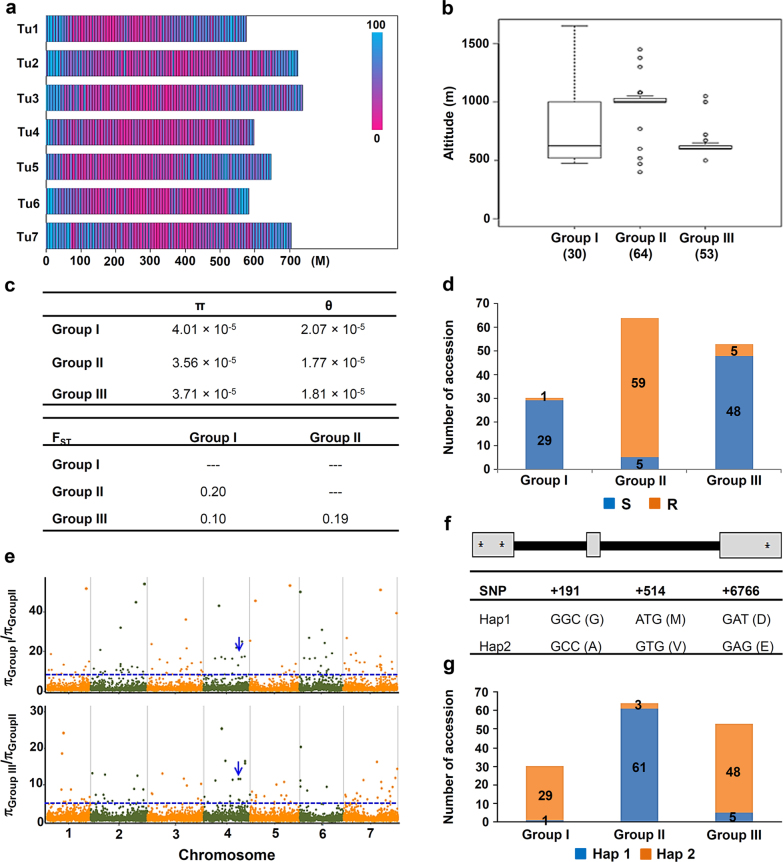
Population analysis. **a**, Distribution of transcriptomic-based SNPs on the seven DNA pseudomolecules (Tu1–Tu7) of *T. urartu*. The SNPs were calculated using a 1-Mb window. **b**, Boxplot comparison of altitude ranges of Tu accessions. The number of accessions in each group is indicated in parentheses. The line inside each box represents the median, the ends of each box define the 25th and 75th percentiles, and the error bars mark the 10th and 90th percentiles. Outliers are displayed as open circles. **c**, Analysis of genetic diversity and differentiation. The *π*, *θ* and *F*_ST_ values were estimated for the three groups of Tu accessions using transcriptomic SNPs. **d**, Reaction phenotypes of Tu accessions to the wheat powdery mildew fungus Bgt. Most of the accessions in Groups I and III (96.7% and 90.6%, respectively) were susceptible to Bgt (race E09), whereas the majority of Group II accessions (92.2%) showed resistance. **e**, A total of 141 (top 1%; *π*_Group I_/*π*_Group II_ > 7.7) and 143 (top 1%; *π*_Group III_/*π*_Group II_ > 4.3) signals were considered to be candidate sweeps (dots above the dashed horizontal threshold line). The Tu accessions in Groups I, II and III were 30, 64 and 53, respectively. Blue arrows indicate the wall-associated receptor protein kinase gene (*TuWAK*, TuG1812G0400002796), whose haplotype variations showed strong associations with resistance or susceptibility to Bgt. **f**, Exon (box)–intron (line) structure of *TuWAK* and its two major haplotypes (Hap1 and Hap2). The positions of the three SNPs that differ between Hap1 and Hap2 are shown. Amino acid changes caused by these SNPs are also displayed. **g**, Distribution of Hap1 and Hap2 in the three groups of *T. urartu* accessions. Hap1 was the major haplotype in Group II, which was strongly associated with resistance to Bgt, while Hap2 was the main haplotype in Groups I and III, and associated with susceptibility to Bgt. In **d** and **g**, the accessions in each group were further sorted based on the response to powdery mildew infection (**d**) or the possession of *TuWAK* haplotypes (**g**), with the assorted accession numbers indicated in appropriate columns.

For population genetic studies, we sequenced the leaf transcriptome of 147 *T. urartu* accessions collected from six countries in the Fertile Crescent (Armenia, Iran, Iraq, Syria, Turkey and Lebanon) (Fig. 3a, Supplementary Data 8, Supplementary Information S3) and identified 144,806 high-quality SNPs from 22,841 expressed genes (Extended Data Fig. 9a). We analysed population structure using STRUCTURE, and showed that based on this or phylogenetic analyses, the Tu accessions clustered into three groups (Fig. 3b, c). Group I contained 30 accessions from multiple countries. Group II contained 64 accessions, 88% of which were from Lebanon. Group III contained 53 accessions, with 92% from Turkey. These groups have differences in the collection site altitudes, with the majority of Group II accessions being from altitudes above 1,000 m (Extended Data Fig. 9b, Supplementary Data 8). The genetic diversity was lowest in Group II (Extended Data Fig. 9c).

After inoculation with the powdery mildew pathogen *Blumeria graminis* f. sp. *tritici*^27^ (Bgt, race E09), 92.2% of the accessions in Group II exhibited resistance, whereas most of the accessions in Groups I and III (96.7% and 90.6%, respectively) were susceptible (Extended Data Fig. 9d). We conducted genomic scans for selective sweeps, and detected 141 (*π*_Group I_/*π*_Group II_ > 7.7) and 143 (*π*_Group III_/*π*_Group II_ > 4.3) candidate sweep signals based on SNP diversity ratios in the compared groups (Extended Data Fig. 9e). These regions included 239 high-confidence predicted genes (Supplementary Data 9), including those with functional annotations for transcriptional regulation (32 genes), signal transduction (14) or detoxification of reactive oxygen species and stress defence (14); 23 of the genes were Tu-specific. Previous studies have found that plant and animal adaptation to high altitudes involves genes with functions in diverse physiological and molecular processes^28,29^. Further analysis showed a wall-associated receptor protein kinase gene (*TuWAK*, TuG1812G0400002796) within a selective sweep signal, which had two haplotypes (Hap1 and Hap2) with differing distributions among the three groups (Extended Data Fig. 9f, g). Hap1 was the major haplotype in Group II, and associated with resistance to Bgt. Hap2 was the main haplotype in Groups I and III, and associated with Bgt susceptibility. The maize homologue *ZmWAK* is involved in defence against fungal pathogens^30^. Therefore, *TuWAK* may have been under selection in Group II accessions, and contributed to both high altitude adaptation and powdery mildew resistance.

## Methods

### Plants

The accession G1812 (PI428198) of *T. urartu* was previously shown by restriction fragment length polymorphism (RFLP) analysis to have the closest relationship to the A subgenome of hexaploid wheat^31^. Its genome was sequenced using a whole-genome shotgun strategy on the Illumina HiSeq2000 platform, and a draft genome has been generated^6^. In this study, we also used accession G1812 of *T. urartu* to improve its genome assembly quality. We combined BAC-by-BAC sequencing with single-molecule real-time (SMRT) sequencing technology (Pacific Biosciences), and new mapping technologies (BioNano genome map and 10× Genomics linked reads) to generate a high-quality reference sequence of *T. urartu*.

### BAC library construction

The genomic BAC libraries of *T. urartu* accession G1812 (PI428198) were constructed by AMPLICON Express (Pullman). In brief, nuclei were isolated from leaves of one-month-old plants, and embedded in agarose plugs. After lysis of nucleic membrane and digestion of proteins, intact high molecular weight (HMW) genomic DNA was extracted as described^32^. The HMW DNA was partially digested using EcoRI, HindIII and MboI to generate insertion DNA fragments for BAC library construction. Subsequently, the DNA fragments (average sizes 120–180 kb) were ligated into the appropriate sites on the vector pCC1BAC or pECBAC1 or pIndigoBAC-5. The ligations were transformed into *Escherichia coli* DH10B cells (phage resistant). Transformants were robotically picked and arrayed onto 384-well plates. All plates were assigned a barcode and recorded in a database. In total, 470,016 BAC clones were obtained, including 184,320 clones in the EcoRI-digest library with an average insert size of 125 kb, 193,536 clones in the HindIII-digest library with an average insert size of 110 kb, and 92,160 clones in the MboI-digest library with an average insert size of 115 kb. The total insert DNA length was about 54.9 Gb, approximately 11 equivalents of the whole genome of *T. urartu*.

### Whole-genome profiling of *T. urartu*

To generate BAC fingerprint contigs, 451,584 BAC clones (179,712 EcoRI clones, 179,712 HindIII clones and 92,160 MboI clones) representing about 10 equivalents of the *T. urartu* genome were analysed using the whole-genome profiling (WGP) technology by Keygene N.V.^33^. After pooling of individual BAC clones, isolation of pooled BAC DNA, digestion with HindIII/MseI, ligation of Illumina Hiseq adaptor sequences and sequencing from the HindIII side using Illumina HiSeq with 100-nt read length, we obtained 1,108,197 filtered WGP tags and 345,233 tagged BACs. Then, the sequence-based physical maps were assembled using an adapted version^33^ of the FingerPrint Contig (FPC) software^34^ to generate a high-stringency map (using a threshold of 1.0 × 10^−28^ DQ) and a reduced-stringency map, in which end-to-end merging of contigs was done and singletons were added in a number of repetitive steps (1.0 × 10^−25^ DQ + end merges 10^−15^ + singleton merges 10^−15^). The total length of BAC contigs reached 5.52 Gb with a BAC contig N50 size of 340 kb for high-stringency WGP map assembly and 4.68 Gb with a BAC contig N50 size of 656 kb for reduced-stringency WGP map assembly.

### Selection of BAC clones and BAC DNA extraction

Using WGP data, 47,200 BAC clones were selected from the tagged 345,233 BAC clones, which were assembled on 20,702 BAC contigs with high-stringency WGP assembly, according to a minimal tiling path (MTP) principle for genomic sequencing via BAC-by-BAC sequencing. The selected MTP BAC clones were divided into two groups (the two neighbouring BACs on each FPC were separated into group A and group B to avoid the misdistribution of sequence reads on BACs after sequencing).

For BAC DNA extraction, each selected BAC clone was incubated in 400 µl lysogeny broth (LB) with 12.5 µg/ml chloramphenicol on 96-well plates at 400 r.p.m. and 37 °C for 16 h. After that, we pooled 2,304 individual BAC clones from 24 pieces of 96-well plates into 96 pools using 2D pooling strategy at horizontal and vertical levels (only BACs from the same group could be pooled together). Each pool contained 48 different BAC clones. For the pooling, 75 µl bacterial solution of each BAC clone was picked up and pooled together as described above for BAC DNA extraction. The BAC DNA was extracted using PhasePrep BAC DNA Kit (Sigma-Aldrich) following the manufacturer’s instructions with some modifications. In brief, bacterial cells were collected by centrifugation for 10 min at 4 °C, and re-suspended in 120 µl chilled resuspension solution containing RNase A solution (20 mg/ml). After addition of 120 µl lysis solution, the bacterial solution was well mixed with the lysis solution by gentle turnover four times, and put on ice for 4 min. Subsequently, 120 µl pre-chilled neutralization solution was added and mixed again by gentle turnover four times. After incubation on ice for 5 min, the lysed bacterial solution was centrifuged at 13,000 r.p.m. and 4 °C for 4 min. Then, the supernatant was transferred to a clean 1.5-ml Eppendorf tube and mixed with 250 µl isopropanol by gentle turnover several times. BAC DNA was pelleted by centrifugation at 13,000 r.p.m. and 4 °C for 20 min. After removal of the supernatant, the pellet was washed using 100 µl 70% ethanol and dried in vacuum. To remove RNAs, we added 500 µl elution solution and 1 µl tenfold diluted RNase cocktail to suspend the pellet. After incubation at 60 °C for 5 min, 20 µl sodium acetate buffer solution and 50 µl endotoxin removal solution were added separately to the DNA solution, mixed and incubated at 37 °C for 5 min. Next, the clean upper phase solution was transferred to a new Eppendorf tube and mixed with 270 µl DNA precipitation solution, and BAC DNA was pelleted by centrifugation at 13,000 r.p.m. and 4 °C for 10 min. Then, the DNA pellet was washed twice using 400 µl 70% ethanol and dried in vacuum. Finally, the BAC DNA was dissolved in 25 µl sterile deionized water, and kept at −20 °C until use. In total, 47,223 BAC clones were pooled into 984 BAC pools and their DNAs were extracted and used for sequencing

### Preparation of Illumina sequencing libraries and sequencing

For sequencing of BACs by Illumina HiSeq2500, we constructed paired-end libraries with ~300 bp insert size following the protocol of NEBNext Ultra DNA Library Prep Kit for Illumina (New England Biolabs) with a slight modification. In brief, 50 ng DNA of each BAC pool (containing 48 BAC clones) was sheared in a Covaris S2 focused ultrasonicator to an average insert size of 300 nt. After end reparation with the NEBNext Ultra End Repair/dA-Tailing Module Kit (New England Biolabs), the DNA fragments were ligated with barcode adaptors, and 96 BAC DNA samples with different barcode adapters were mixed together and purified using a QIAquick PCR Purification Kit (Qiagen). Subsequently, approximately 300-bp DNA fragments were selected again on 2% agarose gel, and amplified by PCR with 11 cycles. The library was then sequenced using 150 base-length read chemistry in a paired-end flow cell on the Illumina HiSeq2500 after library profile analysis by the Agilent 2100 Bioanalyzer and qPCR quantification. Paired-end sequencing was performed following the manufacturer’s protocol (http://www.illumina.com/) based on the workflow: cluster generation, template hybridization, isothermal amplification, linearization, blocking, denaturation and hybridization of sequencing primers. The base-calling pipeline (Hiseq2500) was used to detect bases from the raw fluorescent images. In total, 39 libraries, including 2,347 pools (48 BACs per pool) with 300 bp insert size, were prepared, 33 sequencing lanes were run in Hiseq2500 and 2,102 Gb of raw sequence data was generated (Extended Data Fig. 1b).

### Data quality control

The raw datasets were first filtered by trimming reads with low-quality bases (quality < 2) at the front end and reads with quality <10 at the back end, and by discarding reads with 20% low-quality bases (quality <10) and reads with a length <75 base pairs. Then, we removed the contaminated sequence reads by blasting the sequence reads with the genomes of *E. coli*, mitochondria, chloroplast, and the vector sequence as well human genomic sequence with BWA software^35^ (Burrows-Wheeler Aligner, http://bio-bwa.sourceforge.net/). On average, 20% of reads were aligned to microbial genome; 4.5% of reads were aligned to vector genome; and 0.5% of reads were aligned to chloroplast, mitochondrion and human genomes. Finally, 75% of sequence reads with a total length of 1,471 Gb (about 294× *T. urartu* genome) remained for assembly of the *T. urartu* genome.

To confirm that the sequence reads assigned to each BAC were from the right BAC clone, we blasted sequence reads of each BAC pool against the WGP BAC tags. BAC pools with sequence reads less than 400 Mb were sequenced again to obtain enough sequences for assembly. We also found that sequence reads of a few BAC pools were blasted to two or more neighbour WGP BAC tags owing to BAC clone contamination. Thus, these BAC clones were picked again and their DNA was extracted and resequenced.

### Preparation of PacBio sequencing libraries and sequencing

To enable assembly of complex repeat structure and GC- and AT-rich regions, which are often unassembled or highly fragmented in next generation sequencing (NGS)-based draft genomes, we also performed whole-genome shotgun sequencing using SMRT sequencing technology (Pacific Biosciences). The library preparation and sequencing were done by Nextomics. Sequencing libraries with 20-kb DNA inserts were prepared following the protocol of the PacBio template preparation kit (DNA Template Prep Kit 1.0) and sequenced using Pacific Biosciences RSII instrument. A total of 109 SMRT cells were processed. Subread filtering was performed using Pacific Biosciences SMRT analysis software (v2.3.1) with the parameters (subread length = 50, minimum polymerase read quality = 75, minimum polymerase read length = 50). In total, 97 Gb clean sequence data were obtained with an average subread length of 8.1 kb and an N50 subread length of 11.2 kb (Extended Data Fig. 1b).

Owing to the high error rate of SMRT reads, we constructed a PCR-free paired-end library with 500 bp insert size using the whole-genome DNA of *T. urartu* with the PCR-free protocol (Illumina kit FC-121-3001) and sequenced it by Hiseq2500 with two lanes. In total, 130 Gb whole genome shotgun paired-end reads with 250 bp read length were obtained (Extended Data Fig. 1b). Subsequently, we filtered the low quality reads and contamination reads with bacterial genome and vectors and obtained 107 Gb (21×) clean reads, which was used for error correction of the SMRT reads.

### BAC assembly

For assembly, the pipeline flowchart outlined in Extended Data Fig. 1a was followed. First, the Illumina clean reads in each BAC pool were separately assembled into contigs using MaSuRCA software^36^. Then, Illumina clean reads in each vertical BAC pool were aligned against the contigs in all horizontal BAC pools with BLAT^37^ and vice versa. The reads, which had ≥90% coverage and ≥99% identity, and appeared only once in all crossing BAC pools, were selected as input reads to the BAC at the cross point. Those input reads assigned to each BAC were assembled again using MaSuRCA software to obtain sequence contigs of each individual BAC. At this stage, the total contig length of each BAC reached 125 kb and the contig N50 was 35 kb on average.

Next, 107 Gb of PCR-free Illumina clean reads with 250 bp read length was used to correct the 97 Gb (19.5×) PacBio raw reads using Proovread^38^, yielding 72 Gb (14×) of corrected PacBio reads. To fill gaps between contigs in each BAC, the corrected PacBio reads were aligned to the BAC contigs with BLASR^39^ (parameters identity = 95%, minlength = 1 kb). Subsequently, the sequence contigs of each BAC were connected with the best-aligned PacBio reads using a customized perl script. After this process, 46,374 of 47,223 BACs (98.2%) were assembled into a single contig and 610 BACs into two contigs, and more than 99.4% of BACs showed an assembled sequence length larger than 100 kb. Finally, we connected the BAC sequences iteratively into contigs based on their overlapping relationship within FPC contig, and obtained 5.33 Gb in total length with a contig N50 of 183 kb.

### Assembly of missing regions

Owing to the cleavage bias of restriction enzymes and low genome coverage (8×) of the BAC libraries used for FPC construction, some regions of the genome were missed from the BAC sequences. To assemble the missed regions, we tried to assemble the whole genome using the corrected PacBio reads with Celera Assembler. However, the Celera Assembler encountered an error during the assembly. The main reason might be the low sequencing depth of PacBio reads for such a complex genome. Therefore, we retrieved the corrected SMRT reads and the previously reported sequence contigs of *T. urartu*^6^, which were not covered by the BAC sequences at a minimum sequence identity of 95% and 98%, respectively, for assembly. We assembled them using MaSuRCA^40^ with default parameters. In total, we assembled additional 204 Mb of sequences into contigs, and added them to the genome assembly.

### Scaffolding using BioNano genome map and 10 × genomics linked reads

For construction of a BioNano genome map, 10-day-old seedlings of G1812 were harvested. The DNA isolation, sequence-specific labelling of megabase gDNA for Irys mapping by nicking, labelling, repairing, and staining (NLRS) and chip analysis were performed by Genergy Bio Technology according to the manufacturer’s instructions (BioNano Genomics). In brief, the enzyme Nt.BspQI with an appropriate label density (11.5 labels per 100 kb) was selected and applied to digest long-range DNA fragments. Then, the NLRS number per DNA fragment was determined using the BioNano Irys system. In total, 502 Gb BioNano mapping molecules with an average length of 265.71 kb was collected. After filtering the molecules with a cutoff at a minimum length of 150 kb and 8 labels per molecule, 417 Gb BioNano molecules (83× effective depth) with an average length of 294.95 kb was obtained (Extended Data Fig. 1b). Furthermore, we used autonoise^5^ and other default parameters in IrysSolve tools based on *T. urartu* genome sequences to determine the de novo assembly noise. The RefAligner and Assembler programs in IrysSolve tools were used to assemble these BioNano molecules with initial assembly *P* value of 1 × 10^−10^ and extension/refinement *P* value of 1 × 10^−11^. After the aforementioned processes, 9,112 BioNano genome maps with a total map length of 4.68 Gb were generated. The N50 length of the BioNano genome map was 0.61 Mb and the average map length was 0.54 Mb.

We used BioNano genome maps and the *T. urartu* sequence contigs to generate hybrid maps with initial and final alignment *P* value of 1 × 10^−10^, chimaeric/conflicting *P* value of 1 × 10^−13^ and merging *P* value of 1 × 10^−11^. The sequence contigs that had conflicts with BioNano genome maps were cut into sub-sequences for additional hybrid map generation. We identified only 631 chimaeric contig assemblies. Finally, hybrid sequence scaffolds were generated based on these hybrid genome maps. The overlapping contigs in hybrid scaffolds were aligned using MUMmer^41^ and merged into sequence contigs.

For generation of 10× Genomics linked reads, the leaf DNA of G1812 was extracted from 10-day-old seedlings and used for the construction of 10× Genomics libraries following the manufacturer’s protocol (10× Genomics). Then, we used the chromium system to barcode short fragments onto long DNA molecules (≥50 kb), and HiSeq X Ten to sequence these short fragments into standard Illumina paired-end reads as linked-reads. Short reads from the same long DNA molecule shared the same barcode. In total, 57 Gb Illumina paired-end reads (11× effective depth) were produced. Furthermore, we used the software longranger-2.1.2^4^ to align the linked reads to the scaffolds of *T. urartu*. A mean molecule length of 31.39 kb and mean of 21 linked reads per molecule were obtained. Subsequently, the large_sv_call subprogram in longranger-2.1.2^4^ was used to find connection information for scaffolds in the *T. urartu* genome. After this step, a large number of sequence scaffolds and contigs that were not on BAC FPC and were not connected by BioNano genome maps owing to their short length were connected to longer scaffolds (>100 kb).

Additionally, the previously reported 10-kb and 20-kb mate pair reads^6^ were also used to connect sequence contigs into scaffolds using SSPACE^42^ with default parameters. To minimize errors introduced in the scaffolding step, we only connected the sequence contigs supported by linkage evidence from BioNano and 10× Genomics. The SMRT raw reads were also applied to fill the gap in scaffolds using PBJelly^43^ with default parameters to generate longer sequence contigs.

### Generation of pseudomolecules

To anchor the assembled sequences onto chromosomes, we developed an F2 population containing 475 individuals from a cross between accessions G1812 and G3146. We sequenced this population using the restriction enzyme TaqI-associated DNA sequencing (RAD-seq) method for calling SNPs and constructing a genetic map. In total, 981 Gb Illumina sequences was generated (1,028 Mb per F2 individual on average). These Illumina reads were mapped to our assembled sequences using BWA MEM^35^, and SNPs were called using the GATK pipeline^44^. In total, 3,751,342 SNPs were identified between G1812 and G3146. The SNPs were then filtered using parameters DP (depth) ≥2 and MAF (minor allele frequency) ≥ 0.1. Finally, 430,979 high quality SNPs were selected and used for genotyping the sequenced F2 individuals. Adjacent SNPs were merged together using a sliding window method (window:50 SNPs; step:50 SNPs) into bins. The bins as input markers were grouped into seven linkage groups through the ML (maximum likelihood) algorithm in joinMAP 4.1^45^, and the linkage map of bins was created using the Kosambi model in MSTmap^46^. In total, 22,386 bins were anchored on the seven chromosomes of *T. urartu*. Using the SNP bin markers, 27,587 sequence contigs were anchored onto chromosomes (Supplementary Data 1). We then assigned the assembled scaffolds onto their corresponding positions on the chromosome using the mapping information from SNPs, and generated seven pseudomolecules. Subsequently, adjacent bins with the same genotype within a physical distance of 100 kb were merged into larger bins to remove bins with spurious or missing genotypes. Furthermore, we set an upper limit on the physical distance between two adjacent crossovers of at least 1 Mb, and allowed only at most ten recombination events on each chromosome, and changed the incompatible genotypes that caused spurious double crossover events to missing data. Finally, we recalculated the genetic distances from the recomputed recombination frequencies through the Kosambi mapping function for each linkage group, and obtained a genetic map consisting of 4,506 high-confidence bins. The accumulated genetic linkage distance of the genetic map was 1,444 cM (Extended Data Fig. 1e).

### Assembly evaluation

To evaluate the quality of our assembly, we compared the Tu genome to 12 previously published BAC sequences (Extended Data Fig. 2a) from the *T. urartu* G1812 genome downloaded from NCBI (http://www.ncbi.nlm.nih.gov/nuccore/?term=Triticum+urartu+BAC) using NUCmer (parameter: -mum -mincluster 700) in MUMmer package^41^. Then we drew the dot plot using mummerplot in the same package with default parameters. We identified the repeat sequences using BLAST with *E* value 1 × 10^−10^ against the TREP database (http://botserv2.uzh.ch/kelldata/trep-db/index.html) and PGSB Repeat Element Database (http://pgsb.helmholtz-muenchen.de/plant/recat/) (Extended Data Fig. 2). All of the BAC sequences were nearly completely covered by our assembled pseudomolecules with an average coverage of 98.89% and sequence identity of 99.66%. These results indicate that we have generated a high quality genome sequence of *T. urartu*.

To evaluate the per base error rate, the WGS short reads, including ‘WGS HiSeq PCRFree 2 × 250’ (Extended Data Fig. 1b) and ‘WGS Hiseq 2 × 150 (GenBank accessions SRR124016 ~ SRR124023)’, were aligned to the *T. urartu* genome with BWA MEM. After filtering out the short alignments (<50 bp), we found that 98.56% of the *T. urartu* genome was covered. Meanwhile, 98.45% of reads in ‘WGS HiSeq PCRFree 2 × 250’ and 98.78% of ‘BGI Hiseq 2 × 150’ reads were aligned to the *T. urartu* genome. After removing the aligned reads with identity ≤98%, we used GATK to call variants from the data above and obtained 541,849 SNPs (0.011%, one per 9 kb) and 128,592 indels with a total size of 281,155 bp (0.006% base error).

To evaluate structural chimeric error rate, we compared the *T. urartu* genome contigs and the BioNano genome map by RefAligner program in IrisSolve package from BioNano Genomics (https://bionanogenomics.com/support/software-downloads/), and detected 5,346 collapse/expansion regions (>1 kb) using the SV detect program in the same package. These collapse/expansion regions covered 54.92 Mb (1.13%) of the assembled genome (4.86 Gb), with a maximum length of 124.43 kb, a minimum length of 1,012 bp, and an average length of 10.27 kb. Among them, there were 4,971 collapsed regions covering 51.26 Mb (1.05% of the assembled genome 4.86 Gb) with a maximum length of 124.43 kb, a minimum length of 1,018 bp and an average length of 10.31 kb, and 375 expansions covering 3.67 Mb (0.07% of total assembled genome 4.86 Gb) with a maximum length of 62.36 kb, a minimum length of 1,012 bp and an average length of 9.78 kb. All the data strongly indicate that the quality of our genome assembly is high and reliable.

### Annotation and analysis of repetitive elements

Repetitive sequences and transposable elements in the *T. urartu* genome were identified using a combination of ab inito and homology-based methods at both DNA and protein levels. In brief, an ab inito repeat library for *T. urartu* was predicted using LTR_FINDER v1.0.2^47^, RepeatModeler (v1.0.3) with default parameters. The library was aligned to PGSB Repeat Element Database (http://pgsb.helmholtz-muenchen.de/plant/recat/) to classify the type of each repeat family. For identification of the repeats throughout the genome, RepeatMasker (v3.2.9) was applied with both the ab initio repeat databases and Repbase (http://www.girinst.org/repbase) using the WU-BLASTX search engine. Overlapping transposable elements belonging to the same repeat class were collated and combined. In addition, we annotated the tandem repeats using the software Tandem Repeats Finder (TRF, v4.04)^48^.

Solo-LTR was identified using LTRharvest^49^. A customized script was implemented to identify intact LTR/Copia and LTR/Gypsy retrotransposons. The ClustalW program^50^ was applied to align 5′ and 3′ solo-LTRs to intact LTR elements. The evolutionary distance of the two LTR sequences was estimated using the Kimura two-parameter method embedded in baseml program in PAML^51^. A substitution rate of 1.3 × 10^−8^ mutations per site per year was used to convert evolutionary distance between 5′ and 3′ solo-LTRs to insertion age of retrotransposons^52^. In total, we identified 35,559 and 48,370 intact Copia and Gypsy retrotransposons, respectively. To identify solo-LTRs, we first excluded intact LTR transposable elements from the dataset of Gypsy and Copia LTR-retrotransposons, and aligned all known LTR segments to those damaged LTR-retrotransposons. The transposons that were similar (identity >85%) to a known LTR segment were identified as solo-LTRs. We verified that only 2.28% of our identified LTRs overlapped with BioNano collapse/expansion regions and 1.76% overlapped with PacBio junction regions, indicating that very few LTRs were affected by misassembly of sequences.

Simple sequence repeat (SSR) markers are useful in plant genetic analysis. Therefore, we detected SSR markers within our assembled sequences using MISA software (http://pgrc.ipk-gatersleben.de/misa/misa.html). A total of 486,506 SSRs were identified. Dinucleotide was the most common repeat motif with a frequency of 35.6% (173,125 SSRs), followed by mono- (170,845, 35.1%), tri- (88,008, 18.1%), and hexa-nucleotide (7,145, 1.5%) repeat motifs. The distribution of SSRs is shown on Fig. 1i and Extended Data Fig. 3.

### Annotation and analysis of non-coding RNAs

Non-coding RNAs of *T. urartu*, including rRNAs, tRNAs, miRNAs and snoRNAs, were analysed. We used tRNAscan-s.e. (version 1.23) with eukaryote parameters to predict tRNAs^53^. The miRNA and snoRNA genes were predicted using Infernal software (version 1.0)^54^ to search the genome against the Rfam database (http://rfam.xfam.org/, release 9.1) with default parameters. The rRNA sequences were predicted using BLASTN (*E* value <1 × 10^−5^) to align the known rRNA genes (5S, 5.8S, 18S, and 28S) of both *T. aestivum* and *Arabidopsis* from GenBank to the draft genome. Additionally, lncRNAs were identified with rigorous criteria: (1) transcript length must be longer than 200 bp; (2) transcript must contain no open reading frame (ORF) longer than 50 amino acids; (3) the Coding Potential Calculator (CPC)^55^ was used to predict the coding potential of each transcript, and those with CPC scores >0 were discarded.

### Gene prediction and functional annotation

We predicted a set of genes in the *T. urartu* genome using evidence-based Gramene pipeline^8^ by combining protein, cDNA, EST and RNA-seq evidence. We downloaded 559,967 mRNAs (https://www.ncbi.nlm.nih.gov/nuccore/?term=Triticum+urartu) and 1,283,261 ESTs (https://www.ncbi.nlm.nih.gov/nucest/?term=Triticum) of *T. urartu* from NCBI nucleotide database as same-species cDNAs and same-species ESTs. SwissProt proteins for plants were cleaned up by removing redundant sequences with a minimum threshold of 80% for both identity and coverage, which left us 340,312 sequences as protein evidence. The protein evidence also included 1,795 wheat proteins downloaded from GenBank. The mRNAs and ESTs of monocot species other than wheat were used as cross-species evidence; these were downloaded from NCBI and filtered to remove redundant sequences with a cutoff of 90% for both identity and coverage, which resulted in 548,604 cDNAs and 978,696 ESTs. RNA-seq data from 243 samples (2.47 Tb) of *T. urartu* and bread wheat were downloaded from NCBI and were assembled into contigs using SOAPdenovo-trans v1.03 (http://soap.genomics.org.cn/SOAPdenovo-Trans.html). The assembled contigs were used as same-species EST evidence.

The transcripts predicted from each evidence type were combined using the Gramene pipeline to generate 115,255 potential genes as a raw set, of which the majority were spurious genes with only EST or RNA-seq evidence support. The expression value of each gene in 243 wheat RNA-seq samples was calculated using Cufflinks^56^ (-u). The raw gene set was filtered to generate a core set of 41,507 protein-coding genes by removing transposable element-related genes, pseudogenes and non-coding genes. We simply define a gene to be transposable element-related if its protein has >50 amino acids or >50% of its protein length aligned to the annotated transposons. For pseudogenes, we treated single-exon and multi-exon genes separately. For a single-exon gene, if its protein is fully covered by a multi-exon gene, it is designated as a pseudogene. For multi-exon gene, if its protein is fully covered by another gene, and its protein length is <70% of the latter, and its expression in RNA-seq data is lower than half of the average of all genes, it is designated as a pseudogene. For genes without protein evidence, a minimum cutoff of 50 amino acids was used to distinguish coding from non-coding genes. For single-exon genes with only EST or RNA-seq evidence support, we further filtered out those with protein length <100 amino acids and expression level in RNA-seq data lower than half of the average of all genes.

We categorized 37,516 genes (90.4%) with multiple types of evidence support as high-confidence and 3,991 genes (9.62%) with single type of evidence support as low-confidence. The number of genes supported by each evidence type is summarized in Extended Data Table 1a. Protein domains of each gene were annotated using InterProScan^57^ by searching against publicly available databases, including ProDom (http://prodom.prabi.fr/), Prints (http://www.bioinf.manchester.ac.uk/dbbrowser/PRINTS/), Pfam (http://pfam.xfam.org/), Smart (http://smart.embl-heidelberg.de/), Panther (http://www.pantherdb.org/), Superfamily (http://supfam.org/SUPERFAMILY/), PIR (http://pir.georgetown.edu/) and Prosite (http://prosite.expasy.org/). Overall, 73.80% of the predicted proteins were found to contain InterPro domains. In addition, 55.08% of predicted genes have been classified by gene ontology terms and 11.35% of the genes were mapped to known biological pathways. Gene function was annotated according to the best matched proteins of *B. distachyon* (http://genome.jgi.doe.gov), and rice (http://rice.plantbiology.msu.edu/downloads_gad.shtml) using BLASTP with both minimum identity and coverage of 30% as thresholds (Extended Data Table 1c).

### Segmentally and tandemly duplicated genes

To understand the chromosomal distribution of duplicated genes, we identified segmentally and tandemly duplicated genes in the Tu genome. Segmentally duplicated genes were identified in collinear segments, which contain at least two collinear gene pairs that were not separated by more than two non-collinear genes. Tandemly duplicated genes were paralogues that were located close to each other, and were not separated by more than two genes.

### Orthologous genes between *T. urartu* and other grass genomes

We applied the standard OrthoMCL pipeline^58^ to identify orthologous gene families among five grass species including *T. urartu*, rice, maize, sorghum and *B. distachyon*. The longest protein from each gene was selected, and the proteins with a length less than 30 amino acids were removed. After this step, pairwise sequence similarities between all input protein sequences were calculated using BLASTP with an *E* value cut-off of 1 × 10^−5^. Markov clustering (MCL) of the resulting similarity matrix was used to define the orthologue cluster structure of the proteins, using an inflation value (−*I*) of 1.5 (OrthoMCL default). Then, comparative analysis was performed among *T. urartu*, rice, maize, sorghum and *B. distachyon* (Extended Data Fig. 4a)

### Transcription factor analysis

To identify transcription factors in *T. urartu* genome, we blasted the annotated genes against known plant transcriptional factors collected from the iTAK database (http://itak.feilab.net/cgi-bin/itak/index.cgi). We then assigned these genes to specific transcription factor families using the prediction tool iTAK^14^. In total, 1,779 genes were classified as transcription factors into 68 families. To compare the size of transcriptional factor families among different species of grasses, we collected transcriptional factors in other six cereal genomes from iTAK (http://itak.feilab.net/cgi-bin/itak/index.cgi) including *B. distachyon*, *Oryza sativa*, *Sorghum bicolor*, *Zea mays*, *Aegilops tauschii* and *T. aestivum*, and investigated the enrichment of genes in each transcriptional factor family in all studied species (Supplementary Data 3).

### Prolamin and disease resistance genes

The wheat prolamin gene sequences, including those encoding HMW-GS, LMW-GS, α-, γ-, ω- and δ-gliadin, were used as queries to blast against the *T. urartu* genome sequences with *E* value 1 × 10^−10^, and matched sequences were extracted and manually annotated. Based on the annotation of whole gene set, we selected all of the NB-ARC domain genes and calculated their RPKM value based on the *T. urartu* RNA-seq data after inoculating with the powdery mildew pathogen *B. graminis* f. sp. *tritici*.

### Gene expression profiling in leaf, root and spike of *T. urartu*

To study gene expression profiles, the RNA-seq data of leaves and roots of two-month-old plants and young spikes with 10–12 cm length were used^6^. Poor quality or technical sequences in Illumina paired-end reads (read length 75 bp) were removed using Trimmomatic version 0.35 preprocessing tool^59^. The qualified paired reads were then aligned against the IGDBv1.0 reference transcript sequences using Bowtie 2 version 2.2.6^60^ to find all alignments of a read with no more than two mismatches. Subsequently, gene and isoform abundances were quantified from paired-end RNA-seq data using the RSEM software package^61^. Differentially expressed transcripts or genes with biological replicates were identified by running Bioconductor tools edgeR^62^, which implements a range of statistical methods based on the negative binomial distributions. The differentially expressed genes were partitioned into clusters with dominantly high expression in one of the three tissues by perl script in Trinity^63^.

Furthermore, we identified the functional preference of the dominantly expressed genes in each organ using a perl script included in Trinotate (http://trinotate.github.io/) to extract all Gene Ontology (GO) assignments from the TrEMBL/SwissProt databases, and used Bioconductor package GOseq to perform functional enrichment tests.

To monitor gene expression level along the *T. urartu* chromosomes in the three organs, we applied a window shift size of 5 Mb in a customized perl script. For each pseudomolecule, gene expression distribution among three organs is shown in Fig. 1k and Extended Data Fig. 3.

### Comparison of *T. urartu* genome with BACs of *T. turgidum* and *T. aestivum*

To investigate sequence variation among different A genomes after polyploidization, we aligned the sequences of six BACs (881 kb in total) from the A subgenome of Tt and 11 BACs (1,423 kb in total) from the A subgenome of Ta to the Tu genome using NUCmer (parameter: -mum -mincluster 700) in MUMmer package^41^. Then we drew the dot plot using mummerplot in the same package with default parameters. We identified the repeat sequences using BLAST with *E* value 1 × 10^−10^ against the TREP database (http://botserv2.uzh.ch/kelldata/trep-db/index.html) and PGSB Repeat Element Database (http://pgsb.helmholtz-muenchen.de/plant/recat/) (Extended Data Fig. 5b).

We also compared the TGACv1 A subgenome sequences of hexaploid wheat^19^ to the Tu genome. All scaffolds which were located on Ta7A were compared to Tu7. We performed all-to-all alignment (-minIdentity = 80~99, -minScore = 100, –fastMap) of Tu7 and Ta7A using BLAT^37^. The percentages of homologous segments on Tu7 and Ta7A were calculated using SOAP.COVERAGE^64^ (Extended Data Fig. 5d).

### Comparison of *T. urartu* genome with *T. aestivum* and *Ae. tauschii*

To investigate chromosomal structure variation between *T. urartu* and polyploid wheat, we compared the *T. urartu* genome with the three subgenomes (A, B, and D) of bread wheat (ftp://ftp.ensemblgenomes.org/pub/release-28/plants/fasta/triticum_aestivum/) as well with the D genome of *Ae. tauschii*^15^. Using software MCScanX^65^ with at least three syntenic genes, we identified orthologous blocks and plotted homologous proteins among wheat genomes. Highly similar proteins (coverage of protein length ≥80 and identity ≥80) were obtained using BLAT^37^ (Fig. 2a, b, Extended Data Fig. 5a).

### Collinearity of *T. urartu* versus *B. distachyon, O. sativa* and *S. bicolor*

We identified homologous proteins between *T. urartu* and the other three genomes using BLASTP^66^ with *E* value 1 × 10^−5^, and scanned syntenic blocks consisted of homologous genes among the four genomes using MCScanX^65^ with at least three syntenic genes (Extended Data Fig. 6).

### Evolution of ancient duplicated blocks in *T. urartu*

We performed intragenomic comparison. Using all-against-all blastp and MCScanX^65^ with default parameters in search of collinear paralogous relationships, five obvious collinear blocks were found in *T. urartu*. These blocks were then compared to seven previously published duplicated chromosome pairs in rice (Extended Data Fig. 7).

### Comparisons of DNA and protein sequences between *T. urartu* chromosome 3 (Tu3) and *T. aestivum* chromosome 3B (Ta3B)

Chromosome 3B of hexaploid wheat was completely sequenced and assembled using BAC-by-BAC sequencing strategy^26^. We performed complete and precise comparisons between Tu3 and Ta3B. The sequence of Ta3B was downloaded from the Ensembl website (ftp://ftp.ensemblgenomes.org/pub/release-28/plants/fasta/triticum_aestivum/). To compare the DNA sequence of the two chromosomes, we performed all-to-all alignment (-minIdentity = 80, -minScore = 100, –fastMap) of Tu3 and Ta3B with BLAT^37^. The percentages of homologous segments on Tu3 and Ta3B were calculated using SOAP.COVERAGE^64^ (Extended Data Fig. 8a).

To compare the transposable element insertion date of Tu3 and Ta3B, we identified 5′ and 3′ solo-LTRs of Tu3 and Ta3B retrotransposons using LTRharvest^49^. The evolutionary distance of the two LTR sequences was estimated by using the Kimura two-parameter method^51^. A substitution rate of 1.3 × 10^−8^ mutations per site per year was used to convert evolutionary distance between 5′ and 3′ solo-LTRs to transposable element insertion dates^52^ (Extended Data Fig. 8h).

### Collinearity between Tu3 and Ta3B

We plotted homologous DNA sequences between Tu3 and Ta3B using NUCmer (parameter: -mum -mincluster 700) in MUMmer package^41^. Collinearity in both regions of 0–200-Mb and 400–700-Mb segments can be clearly observed between two chromosomes. Within 200–400-Mb segments, collinearity was not identified between Tu3 and Ta3B.

We also explored collinear relationships of homologous genes between Tu3 and Ta3B. Syntenic blocks containing at least three homologous gene pairs were identified using MCScanX^65^ with at least three syntenic genes (Extended Data Fig. 8b–g).

### Identification of gene insertions and deletions on Tu3 and Ta3B

We computationally identified gene insertions and deletions on Tu3 relative to Ta3B by combining data from *B. distachyon* (Bdistachyon_283_v2.1), rice (IRGSP1.0) and sorghum (http://phytozome.jgi.doe.gov/pz/portal.html). They are grass relatives of wheat that have well-sequenced and annotated genomic data.

MCScanX^65^ was used to identify collinear duplicated gene blocks between species or genomes, including Tu3-to-Ta3B, Tu3-to-Bd, Tu3-to-Os, Tu3-to-Sb, Ta3B-to-Bd, Ta3B-to-Os and Ta3B-to-Sb. We collected 176 syntenic blocks between Tu3 and Ta3B, each containing more than five paired orthologues. Customized perl scripts were used to identify gene insertions and deletions occurred in the syntenic blocks between Tu3 and Ta3B via detecting whether the orthologues in Bd, Os and Sb exist. If a Ta3B gene had orthologues in at least two of Bd, Os and Sb but not on Tu3, a gene deletion was defined to have occurred on Tu3. A Tu3 insertion was defined if no collinear orthologues were found in any other investigated genomes, and the orthologues of two adjacent genes around the inserted segment existed in a collinear region of at least two of Bd, Os and Sb.

### Analysis of *T. urartu* populations

A total of 147 *T. urartu* accessions, collected from Armenia, Iran, Iraq, Syria, Turkey and Lebanon, were used in this study (Supplementary Data 8). Leaf samples from five uniform seedlings were used for total RNA extraction with Illumina TruSeq RNA Sample Prep Kit. With them, 147 paired-end libraries were constructed using Illumina Paired-End Sample Prep Kit, and sequenced by Illumina HiSeq 2000 platform. In total, 63 billion paired-end reads in length of 100 bp were generated (6.3 Tb of sequences), with an average coverage depth of more than 25× for each accession. Then, adaptor sequence trimming and removal of low-quality reads were performed with the ngsShoRT algorithms^67^.

After removing adaptor sequences and reads with low sequence quality, TopHat2^68^ was used to map the paired-end reads against the reference sequence of G1812. Only paired-end reads that mapped uniquely to the genome were used for further analysis of variation calling. Duplicated reads were also filtered. The SNP calling were performed by SAMtools mpileup package^69^, and SNPs with minor allele frequency lower than 5% were excluded from further analyses.

For population structure analysis, the neighbour-joining tree was constructed using MEGA5 based on all of the SNPs^70^. Population structure was calculated using STRUCTURE software^71^. The number of genetic clusters *K* was predefined as 1–10 to explore the population structure with three iterations. The run with maximum likelihood was used to assign individual genotypes into groups. The three groups uncovered using STRUCTURE corresponded well to those based on phylogenetic clustering with respect to accession composition in each group. The statistics of sequence diversity (*F*_ST_) and the population differentiation (*π* and *θ*) were computed using a 100-kb window in 10-kb steps with the PopGen package in BioPerl^72^.

### Reporting summary

Further information on experimental design is available in the Nature Research Reporting Summary linked to this paper.

### Data availability

Sequence data and assemblies have been deposited at BioProject under project accession number PRJNA337888, Sequence Read Archive SRP081049 (PacBio reads SRR4010673–SRR4010781 and Illumina PCR-free reads SRR4010671–SRR4010672), and GenBank MKGO00000000 (pseudomolecules of *T. urartu*). The Tu genome annotation is available at MBKBase website (http://www.mbkbase.org/Tu/). BAC assemblies and the sequence reads of the population (including SNPs) have been deposited at GSA (http://gsa.big.ac.cn/) with the accession number PRJCA000369.

## Online content

Any Methods, including any statements of data availability and Nature Research reporting summaries, along with any additional references and Source Data files, are available in the online version of the paper at 10.1038/s41586-018-0108-0.

### Supplementary information

Supplementary Information This file contains Supplementary Information sections 1-4, Supplementary Figures 1-2 and Supplementary Tables 1-2.

Reporting Summary

Supplementary Data 1 This file contains an assignment of assembled sequence contigs on the chromosomes of *T. urartu*.

Supplementary Data 2 This file contains a comparison of the Tu genome sequence with BAC sequences from A genome of *T. turgidum* (Tt) and *T. aestivum* (Ta).

Supplementary Data 3 This file contains a comparison of transcription factors among 7 sequenced grasses.

Supplementary Data 4 This file contains the R genes predicted in the *T. urartu* genome and their location and expression abundance.

Supplementary Data 5 This file contains a list of prolamin genes.

Supplementary Data 6 This file contains the synteny between *T. urartu* (Tu) and *B. distachyon* (Bd), *O. sativa* (Os) and *S. bicolor* (Sb).

Supplementary Data 7 This file contains gene insertions and deletions identified between Ta3B and Tu3.

Supplementary Data 8 This file contains information on 147 *T. urartu* accessions used for population analysis.

Supplementary Data 9 This file contains a list of 239 high confidence (HC) genes corresponding to the significant selection sweep signals.

## Figures and Tables

**Extended Data Table 1:**
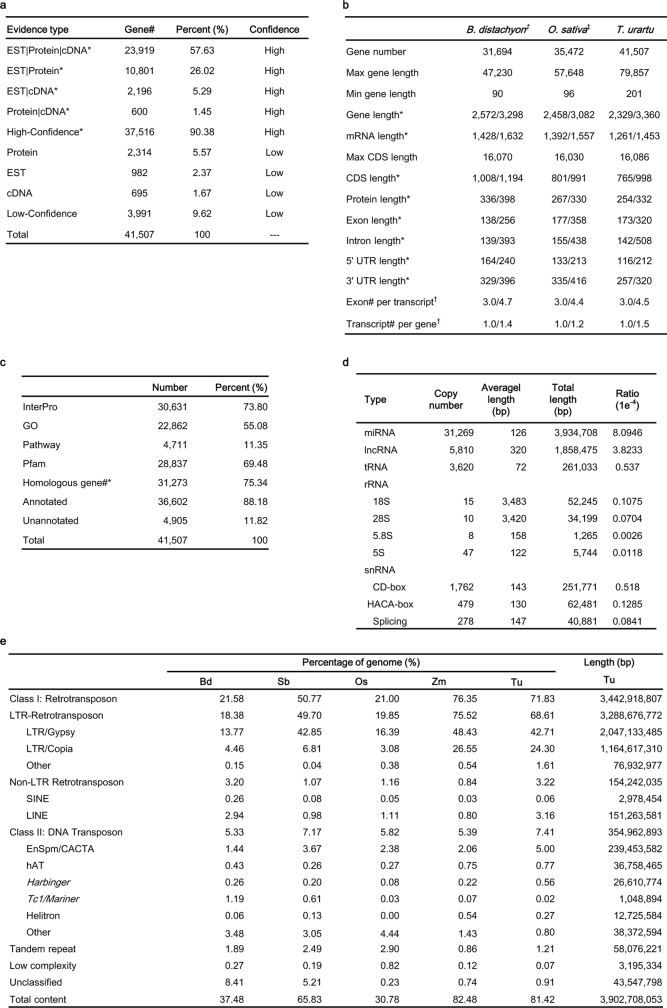
Summary of genome annotations of *T. urartu*

**a**, The number of genes supported by each evidence type in core gene set of *T. urartu*. *Vertical bar indicates multiple types of evidence support. Cross-species evidence was categorized as protein evidence. **b**, Gene numbers and features of *T. urartu*. *The median/average length in base pair (bp). †The median/average in number. ‡*B. distachyon*: Bdistachyon_283_v2.1 gene set. *O. sativa*: RAPV1 gene set. **c**, Functional annotation of the predicted genes in *T. urartu*. *Based on comparison with *B. distachyon* and *O. sativa*. **d**. Summary of predicted non-coding RNAs in the genome of *T. urartu*. **e**, Comparison of repetitive DNA content among *T. urartu* and four other sequenced grasses. Bd, *B. distachyon*; Sb, *S. bicolor*; Os, *O. sativa*; Zm, *Z. mays*, Tu, *T. urartu*.
