# Supplementary material for: Genome sequence of the progenitor of wheat A subgenome Triticum urartu
Source: Nature. 2018 May 9;557(7705):424–8. doi: 10.1038/s41586-018-0108-0 (PMC6784869; doi:10.1038/s41586-018-0108-0)
Supplement: Supplementary file 1 — This file contains Supplementary Information sections 1-4, Supplementary Figures 1-2 and Supplementary Tables 1-2. [file 41586_2018_108_MOESM1_ESM.pdf]

nature

SUPPLEMENTARY INFORMATION

https://doi.org/10.1038/s41586-018-0108-0

In the format provided by the authors and unedited.

Genome sequence of the progenitor of wheat A subgenome *Triticum urartu*

Hong-Qing Ling[1,2,6]*, Bin Ma[3,6], Xiaoli Shi[1,6], Hui Liu[3,6], Lingli Dong[1,6], Hua Sun[1,6], Yinghao Cao[3], Qiang Gao[3], Shusong Zheng[1], Ye Li[1], Ying Yu[3], Huilong Du[2,3], Ming Qi[3], Yan Li[3], Hongwei Lu[2,3], Hua Yu[3], Yan Cui[1], Ning Wang[1], Chunlin Chen[1], Huilan Wu[1], Yan Zhao[1], Juncheng Zhang[1], Yiwen Li[1], Wenjuan Zhou[1], Bairu Zhang[1], Weijuan Hu[1], Michiel J. T. van Eijk[4], Jifeng Tang[4], Hanneke M. A. Witsenboer[4], Shancen Zhao[5], Zhensheng Li[1], Aimin Zhang[1]*, Daowen Wang[1,2]* & Chengzhi Liang[2,3]*

[1]State Key Laboratory of Plant Cell and Chromosome Engineering, Institute of Genetics and Developmental Biology, Chinese Academy of Sciences, Beijing, China. [2]College of Life Sciences, University of Chinese Academy of Sciences, Beijing, China. [3]State Key Laboratory of Plant Genomics, Institute of Genetics and Developmental Biology, Chinese Academy of Sciences, Beijing, China. [4]Keygene N.V., Wageningen, The Netherlands. [5]BGI-Shenzhen, Shenzhen, China. [6]These authors contributed equally: Hong-Qing Ling, Bin Ma, Xiaoli Shi, Hui Liu, Lingli Dong, Hua Sun.
*e-mail: hqling@genetics.ac.cn; amzhang@genetics.ac.cn; dwwang@genetics.ac.cn; cliang@genetics.ac.cn

© 2018 Macmillan Publishers Limited, part of Springer Nature. All rights reserved.

SUPPLEMENTARY INFORMATION

Genome sequence of the progenitor of wheat A subgenome *Triticum urartu*

Hong-Qing Ling[1,2*#], Bin Ma[3*], Xiaoli Shi[1*], Hui Liu[3*], Lingli Dong[1*], Hua Sun[1*], Yinghao Cao[3], Qiang Gao[3], Shusong Zheng[1], Ye Li[1], Ying Yu[3], Huilong Du[2,3], Ming Qi[3], Yan Li[3], Yan Cui[1], Ning Wang[1], Chunlin Chen[1], Huilan Wu[1], Yan Zhao[1], Juncheng Zhang[1], Yiwen Li[1], Wenjuan Zhou[1], Bairu Zhang[1], Weijuan Hu[1], Hongwei Liu[2,3], Michiel J.T. van Eijk[4], Jifeng Tang[4], Hanneke M.A. Witsenboer[4], Shancen Zhao[5], Zhensheng Li[1], Aimin Zhang[1#], Daowen Wang[1.2#], Chengzhi Liang[2,3#]

[1]State Key Laboratory of Plant Cell and Chromosome Engineering, Institute of Genetics and Developmental Biology, Chinese Academy of Sciences, Beijing, China

[2]College of Life Sciences, University of Chinese Academy of Sciences, Beijing, China.

[3]State Key Laboratory of Plant Genomics, Institute of Genetics and Developmental Biology, Chinese Academy of Sciences, Beijing, China

[4]Keygene N.V., Wageningen, the Netherlands

[5]BGI-Shenzhen, Shenzhen, China

[*]These authors contributed equally to this work

[#]Corresponding authors

Content

S1 Analyses of gene families and gene expression **3**

S1.1 Transcription factor analysis 3

S1.2 Families of disease resistance and prolamin genes 3

S1.3 Gene expression profiling in leaf, root and spike of *T. urartu* 4

S2 Comparative genomics analysis **5**

S2.1 Comparison of *T. urartu* genome with other wheat genomes 5

S2.2 Genomic comparison of *T. urartu* (Tu) with *O. sativa* (Os), *B. distachyon* (Bd) and *S. bicolor* (Sb) 6

S2.2.1 Collinearity of *T. urartu* versus *B. distachyon*, *O. sativa* and *S. bicolor* 6

S2.2.2 Reconstruction of *T. urartu* chromosomes from 12 ancestral chromosomes 7

S2.3 Evolution of ancient duplicated blocks in *T. urartu* 8

S2.4 Comparison of chromosome 3 of *T. urartu* (Tu3) with chromosome 3B of *T. aestivum* (Ta3B) 10

S2.4.1 Comparisons of DNA and protein sequences between Tu3 and Ta3B 10

S2.4.2 Collinearity between Tu3 and Ta3B 11

S2.4.3 Identification of gene insertions and deletions on Tu3 and Ta3B 12

S3 Analysis of *T. urartu* populations **12**

S3.1 RNA-seq reads mapping and SNP calling 12

S3.2 Population genetic analysis 12

S4. References **13**

S1 Analyses of gene families and gene expression

S1.1 Transcription factor analysis

Statistical tests showed that the gene number of the most transcriptional factor families was relatively conserved among the 7 genomes with an exception of transcriptional factor B3 family (**Supplementary Data 3**). The gene number of the B3 family was significantly increased in *T. urartu*, *Ae. tauschii* and *T. aestivum* in comparison to that in *B. distachyon, O. sativa, S. bicolor* and *Z. mays*. By identifying orthologues of these B3 transcriptional factors in *Arabidopsis* (Luo et al., 2013, Swaminathan et al., 2008), we assigned the B3 transcriptional factors of *T. urartu* to four subfamilies, AUXIN RESPONSE FACTOR (ARF), RELATED TO ABI3 and VP1 (RAV), REPRODUCTIVE MERISTEM (REM) and LEAFY COTYLEDON2 [LEC2]–ABSCISIC ACID INSENSITIVE3 [ABI3]–VAL (LAV). Interestingly, the enlarged number of B3 transcription factors in the three wheat genomes is mainly caused by the expanded REM subfamily (**Extended Data Figure 4c**). It was reported that the REM subfamily functions preferentially in flower development and vernalization (Luo et al., 2013). Thus, dominant B3 transcription factors in wheat genome may be related to the adaption of cold season and involved in the processes of vernalization and flower development. However, more experiments are needed to confirm it.

S1.2 Families of disease resistance and prolamin genes

Given the report that there was a specific expansion of R genes (disease resistance genes) in the *T. urartu* genome (Ling et al., 2013), we also conducted comparison on *R* genes. A total of 598 genes that encode NB-ARC domain and disease resistance proteins were identified, compared to 593 R genes which were detected in the draft genome of *T. urartu* (Ling et al., 2013). The chromosomal locations of the R genes are shown in **Supplementary Data 4**. Among the 598 R genes, only seven genes (1.2%) were not completely sequenced, and contained ‘N’s in their ORF or the 1 kb promoter sequence, whereas 36.3% of *R* genes reported in the draft sequence (Ling et al., 2013) carried with N bases. These results further support the improved quality and completeness of our new assembly. This information will facilitate the functional studies of *R* genes in *T. urartu* and their applications in improving the disease resistance of wheat.

In bread wheat, prolamin proteins play a major role in controlling the end-use quality of grains. The genes encoding prolamins are carried mainly in *Glu-1* (specifying high-molecular weight glutenin subunits), *Glu-3* (specifying low-molecular weight glutenin subunits), *Gli*-1 (specifying γ- and ω-gliadins) and *Gli-2* (specifying α-gliadins) loci. In our *T. urartu* genome sequence, the loci orthologous to *Glu-1* (i.e., *Glu-A1*), *Glu-3* (*Glu-A3*), *Gli-1* (*Gli-A1*) or *Gli-2*

(*Gli-A2*) were well assembled. Two high-molecular weight glutenin subunit genes were present in *Glu-A1*. Four active genes, encoding i-type low-molecular weight glutenin subunits, were found in *Glu-A3*. In *Gli-A1*, five genes encoding one γ- and four ω-gliadins were annotated. In *Gli-A2*, 11 α-gliadin genes were detected (**Supplementary Data 5**). The organization and gene numbers observed at the *Glu-A1*, *Glu-A3* and *Gli-A1* loci of *T. urartu* are well consistent with previous studies on corresponding loci in bread wheat and the D genome donor *Ae. tauschii* (Dong et al., 2016, Dong et al., 2010) and comparable to the annotations in the A subgenome of the common wheat land race Chinese Spring (CS) by Clavijo *et al.* (2017) (two HMW-GS, four LMW-GS, six γ-gliadin, three ω-gliadin, and 10 α-gliadin genes). These results present a valuable reference for future studies on this important locus in bread wheat and related grasses.

S1.3 Gene expression profiling in leaf, root and spike of *T. urartu*

We identified 61,145 transcripts using RNA-seq data of three *T. urartu* tissues leaf, root and spike. Of them, 5,944 (9.7%), 3,884 (6.4%) and 5,483 (9.0%) revealed a differential expression (FDR < 1e-4) between leaf and root, between leaf and spike as well as between spike and root, respectively. The differentially expressed genes were partitioned into clusters with dominantly high expression in spike (**Supplementary Information Figure S1a**), leaf (**Supplementary Information Figure S1b**) and root (**Supplementary Information Figure S1c**), respectively. Gene ontology analysis of genes within each group shows organ specificity in gene functions. Genes that are preferentially expressed in spike are enriched for GO terms relative to hydrolase activity, polysaccharide, carbohydrate, fatty acid and lipid metabolic processes (**Supplementary Information Figure S1a**); leaf specifically expressed genes are enriched for photosynthesis, pigment, chlorophyll and tetrapyrrole metabolic processes (**Supplementary Information Figure S1b**); and root specific genes are enriched for oxidoreductase, peroxidase and antioxidate activity (**Supplementary Information Figure S1c**).

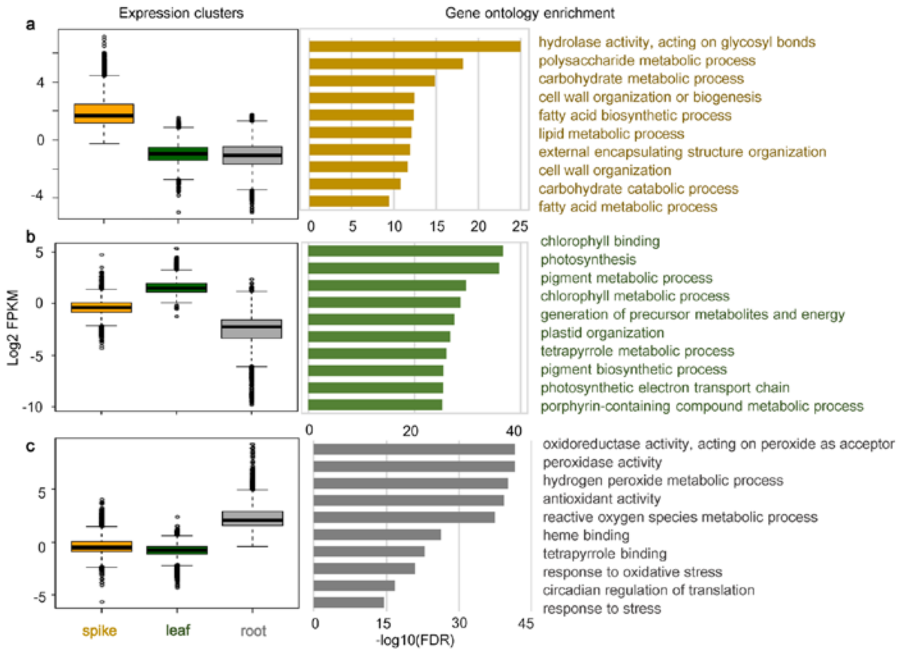

Supplementary Information Figure 1. Gene expression level in three organs of *T. urartu*. Left panels display the expression clusters of three groups of genes preferentially highly expressed in spike (n = 3,170 genes; boxplots minima (from left to right) =-0.23, -4.95, -4.97; maxima = 7.12, 1.52, 1.77; medians = 1.66, -0.94, -1.05; percentiles (75%) = 2.47, -0.49, -0.47); leaf (n = 2,915 genes; boxplots minima (from left to right) = -4.36, -1.27, -9.85; maxima = 4.74, 5.27, 2.35; medians = -0.37, 1.48, -2.23; percentiles (75%) = 0.06, 1.97, -1.56) and root (n = 3,007 genes; boxplots minima (from left to right) = -5.66, -4.33, -0.44; maxima = 3.96, 2.37, 9.21; medians = -0.51, -0.77, 2.06, percentiles (75%) = 0.02, -0.45, 2.88), respectively. Right panels show gene ontology analysis of the three groups.

S2 Comparative genomics analysis

S2.1 Comparison of *T. urartu* genome with other wheat genomes

As shown in **Figure 2a and 2b and Extended Data Figure 5a**, the 7 pseudomolecules of *T. urartu* (Tu) showed a good collinearity with the A, B and D subgenomes of *T. aestivum* (Ta). Meanwhile, we detected several structure variations (segmental inversions and translocations) during wheat genome evolution: (1) We discovered that ~573-605 Mb region of Tu4 is syntenic to Ta5BL/Ta5DL and ~605-649 Mb region of Tu5 syntenic to Ta4BL/Ta4DL, whereas they are clearly collinear with the corresponding parts of Ta4A and Ta5A. Similar phenomena were also found in comparison between *T. urartu* and *Ae. tauschii* (**Extended Data Figure 5a**). The results support that a reciprocal translocation should be occurred at the distal end of long arms between Tu4 and Tu5, and the translocation event happened after the

divergence of A, B and D genomes and before tetraploidization of A and B genomes. Similar observed results were reported by Miftahudin et al. (2004) and Ma et al. (2015). (2) We also observed that a fragment at end Tu7, which showed a good synteny with the corresponding part of Ta7A, Ta7D and chromosome 7 of *Ae. tauschii*, but not with the corresponding fragment of Ta7B, displayed a synteny with the end part of Ta4A in comparison of Tu with Ta (**Figures 2a and 2b, Extended Data Figure 5a**). Based on the results, it is reasonable to deduce that the distal segment localized on Ta4A should come from a one way translocation from Ta7B, which is similar with the reports (Miftahudin et al., 2004, Ma et al., 2015, Clavijo et al., 2017). This translocation event occurred during/after polyploidization of A and B genomes. The novel translocations found in TGACv1 hexaploid wheat genome (Clavijo et al., 2017) were not identified in Tu genome. (3) Another obvious genome structure variation was observed between Tu4 and Ta4A, where Tu4AL corresponds to Ta4AS and Tu4AS to Ta4AL (**Figures 2a and 2b**), while such variation was not determined in comparison of Tu4 with TaB and TaD genomes. This result indicates that a pericentric inversion involving most of the long and short arm occurred on Ta4A during the evolution of Ta4A chromosome. This inversion occurred during or after the generation of tetra- or hexaploid wheat, since it was only found on Ta4A.

S2.2 Genomic comparison of *T. urartu* (Tu) with *O. sativa* (Os), *B. distachyon* (Bd) and *S. bicolor* (Sb)

S2.2.1 Collinearity of *T. urartu* versus *B. distachyon, O. sativa* and *S. bicolor*

The evolutionary relationships between wheat and several other genome-sequenced grasses including *Brachypodium*, sorghum and rice had been reported (The International Brachypodium Initiative, 2010). Among them, *Brachypodium* is the closest and sorghum the farthest relative of wheat. The former diverged with wheat about 32-39 MYA and the latter about 45-60 MYA. Rice split with wheat about 40-54 MYA.

We found that Tu3 and Tu6 were two mostly conserved chromosomes. Tu3 shared common ancestor with Os1-Bd2-Sb3, while Tu6 with Os2-Bd3-Sb4. Notably, syntenic regions of consecutive Tu chromosomal segments were separated by non-homologous DNA segments with varied length in Bd, Os and Sb. For Tu3, two collinear blocks were separated by non-collinear segment of 12-40 Mb on Bd2, 10-20 Mb on Os1 and 12-48 Mb on Sb3. As for Tu6, two collinear regions were divided by 8-47 Mb on Bd3, 9-20 Mb on Os2 and 13-49 Mb on Sb4. The results suggest that segmental deletions likely occurred on Tu3 and Tu6 after divergence between Tu and Bd (**Figure 2c, Extended Data Figure 6, Supplementary Data 6**).

The second conserved chromosomes were Tu1, Tu2, Tu4 and Tu7, which comprised of two chromosomal segments originated from different ancient chromosomes. The majority of Tu1 were orthologous with Os5-Bd2-Sb9. Homologous segments of Os10-Bd3-Sb1 inserted into it. About 400 Mb segment (from 20 to 420 Mb) of the Tu2 shared common ancestor with Os7-Bd1-Sb2, and two segments around it corresponded to Os4-Bd5-Sb6. The smaller segment was about 20 Mb and the larger one was from ~420 to ~740 Mb. Similarly, most chromosomal regions of Tu4 and Tu7 were orthologous with Os3-Bd1-Sb1 and Os6-Bd1-Sb10, respectively. Internal parts from ~51 to ~141 Mb on Tu4 were homologous segments of Os11-Bd4-Sb5 and from ~168 to ~470 Mb on Tu7 were homologous segments of Os8-Bd3-Sb7 (**Figure 2c, Extended Data Figure 6, Supplementary Data 6**)

The least conserved *T. urartu* chromosome was Tu5. It was derived by concatenation of segments originated from three different ancestor chromosomes. Sequentially, segment from 0 to ~317 Mb on Tu5 was homologous to Os12-Bd4-Sb8; segment from ~317 to ~530 Mb corresponded to Os9-Bd4-Sb2 and segment from ~530 to ~648 Mb corresponded to Os3-Bd1-Sb1 (**Figure 2c, Extended Data Figure 6, Supplementary Data 6**). This observation is consistent with the model described by Pont et al. (2013).

S2.2.2 Reconstruction of *T. urartu* chromosomes from 12 ancestral chromosomes

Based on the report that rice well maintained the basic structure of 12 chromosomes of grass ancestor (Salse et al., 2008), we reconstructed the chromosomal evolution model of *T. urartu* from the 12 ancestral chromosomes (A1-A12) using the precise collinear relationships between *T. urartu* and rice. The *T. urartu* chromosomes were mostly formed by insertion of one ancestral chromosome into centromeric region of another. Tu1 was formed by insertion of A10 into A5 (corresponding to Os10 and Os5), Tu2 by insertion of A7 into A4 (corresponding to Os7 and Os4), Tu4 by insertion of A11 into A3 (corresponding to Os11 and Os3) and Tu7 by insertion of A8 into A6 (corresponding to Os8 and Os6). Tu5 is an exception. It was derived from concatenation of A12 and A9 (corresponding to Os12 and Os9). Moreover, segments from two distal ends of A3 joined with A9 to build a complete Tu5 (**Figure 2c**). There were two inversions and three translocations in the formation of *T. urartu* genome. The fusion models of *T. urartu* chromosomes from ancestral chromosomes are completely different from that of *B. distachyon* (The International Brachypodium Initiative, 2010), indicating that the chromosome evolution of *T. urartu* (even Triticeae) must be independent from that of *B. distachyon*.

To perform more accurate and detailed investigation of the evolutionary scenario of *T. urartu* at gene level, we inferred 11,718 *T. urartu* AGK (ancestral grass karyotype) genes via

identifying orthologues from the 14,241 AGK genes defined by Murat et al. (2017). AGK genes in *T. urartu* accounted for 31.2% of all chromosome localized genes. This was lower than the percentages detected in rice (32.4%) and *Brachypodium* (47.4%) (Murat *et al*., 2017). These AGK genes were depleted in pericentromeric and subtelomeric regions (**Figure 2c**), indicating that more new genes are likely to occur in these regions of *T. urartu* genome.

With above mentioned model of chromosome reconstruction, we accurately localized the loci of chromosomal fusions of *T. urartu* and investigated the relationships between localizations of chromosomal fusions and AGK genes. We observed that fusion locations were preferentially in the non-AGK gene-rich regions (**Figure 2c**). Chi-square test also supported that AGK genes significantly depleted at fusion locations (p-value = 0.02). Thus non-AGK genes-rich regions have more chance to occur chromosomal structure variations in *T. urartu* evolution.

S2.3 Evolution of ancient duplicated blocks in *T. urartu*

The common ancestor of grasses has undergone a whole genome duplication (WGD) and subsequent events including chromosome translocations, fusions and insertions to shape the structure of extant various grass genomes. Seven ancestral chromosomes were doubled into 14 chromosomes and subsequent two chromosomal fusions formed a 12-chromosome ancestor. Ancestral duplicated chromosomes were majorly maintained in rice. They are Os1-Os5, Os2-Os4, Os2-Os6, Os3-Os7, Os3-Os10, Os8-Os9 and Os11-Os12 (Salse et al., 2008). Five duplication blocks were identified based on an intra-specific comparison of *T. urartu* genome.

The largest duplication block is between Tu1 and Tu3 covering the chromosomal regions from 434-557 Mb on Tu1 and 389-634 Mb on Tu3 (**Extended Data Figure 7f**). Synteny analysis of Tu3 vs. Os1 and Tu1 vs. Os5 showed that large segments of these two groups of chromosomes were collinear. The duplicated block between Tu1 and Tu3 corresponds to rice duplicated block between Os1 and Os5 (**Extended Data Figures 8a and 7f**). We identified 693 syntenic genes between Os1 and Os5. Of them, 310 (45%) and 320 (46%) genes of Os1 and Os5 have syntenic orthologues on Tu3 and Tu1, respectively, while only 147 (21%) genes are paired in *T. urartu* (**Extended Data Figure 7g**).

Another significant *T. urartu* block is between Tu2 and Tu6. It corresponds to the rice duplication block between Os2 and Os4 (**Extended Data Figures 7d and 7f**). There are 378 syntenic paralogues between Os2 and Os4. Of them, 107 (28%) and 206 (54%) genes of Os2 and Os4 have syntenic orthologues on Tu6 and Tu2, respectively, where 60 (16%) genes are

paired in *T. urartu* (**Extended Data Figure 7g**).

Other two *T. urartu* duplication blocks are between Tu6 and Tu7. One located on 2-141 Mb and 578-680 Mb of Tu6 and Tu7, the other on 471-548 Mb and 78-172 Mb of Tu6 and Tu7 (**Extended Data Figures 7d and 7f**). Synteny analysis of Tu6 vs. Os2 and Tu7 vs. Os6 indicates that the two *T. urartu* blocks correspond to rice duplication between Os2 and Os6 (**Extended Data Figure 7d**). There are 425 syntenic paired-genes between Os2 and Os6. About 22% of the gene pairs are maintained in the collinear blocks of Tu6 and Tu7 (**Extended Data Figure 7g**).

The last *T. urartu* duplicated block between Tu5 and Tu7 (Segments 346-523 Mb on Tu5 and 201-282 Mb on Tu7) corresponds to rice duplication between Os8 and Os9 (**Extended Data Figures 7b and 7f**). About 10% of 305 gene pairs in rice block between Os8 and Os9 were maintained in the syntenic block between Tu7 and Tu5 (**Extended Data Figure 7g**).

In addition, counterparts of three rice duplication pairs, Os3-0s7, Os3-Os10 and Os11-Os12, are not visible in *T. urartu*. Collinearity between *T. urartu* and Os3, Os7 and Os10 is significant, but the corresponding *T. urartu* pairs fail to show significant intra-specific collinearity (**Extended Data Figures 7e and 7f**). Surprisingly, a highly conserved duplication between Os11 and Os12 in rice was lost in *T. urartu*. Almost none of paired-genes in the duplication between Os11 and Os12 are maintained in *T. urartu* (**Extended Data Figure 7c**). These observations indicated that Tu4, which was formed by insertion of A11 into A3, and Tu5, which was formed by concatenation of A12, A9 and segments from A3, were subjected to more variations in wheat evolution.

Rice and *T. urartu* inter-specific collinearity showed that, except for a segment on Os10, both copies of rice syntenic chromosomal segments exist in *T. urartu* genome. Observable Tu syntenic blocks generally retained more collinear gene pairs than regions missing intra-specific collinearity (**Extended Data Figure 7g**). Within 2,620 syntenic paired-genes produced by intra-specific duplication of rice, 2,231 lost single or double copy in *T. urartu*. More than 47% of paired-gene losses in *T. urartu* were caused by missing of single gene copy. *T. urartu* counterparts of Os11 and Os12 were Tu4 and Tu5, respectively. Synteny between Os11 and Os12 is invisible between Tu4 and Tu5. It lost almost all syntenic paired-genes in *T. urartu* and 63% were double-copy losses. Two corresponding segments were close to centromere with sparse genes in *T. urartu* and two telomeric segments with dense genes in rice genome (**Extended Data Figure 7c)**. Severe damage of this collinearity might be due to alteration of chromosomal localization and accumulation of massive transposable elements during speciation of the Triticeae. Overall, only a small portion of collinearity formed by the

WGD of common ancestor of grasses was maintained in *T. urartu*. No large scale segmental or whole genome duplications were found in *T. urartu* genome since its divergence from the common ancestor of grasses.

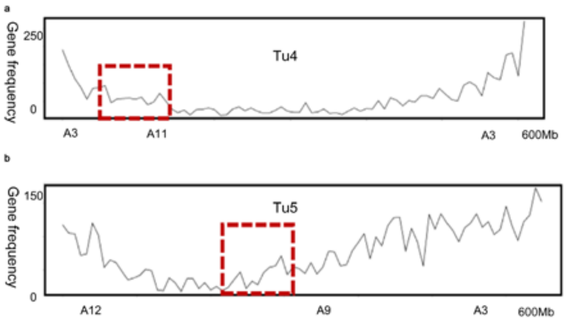

Supplementary Information Figure S2: Gene frequency distribution at ancient duplicated segments between Tu4 and Tu5. Tu4 is comprised of A3 and A11 and Tu5 is comprised of A12, A9 and A3. Ancestral duplicated segments corresponding to duplication between Os11 and Os12 are displayed by red rectangles.

S2.4 Comparison of chromosome 3 of *T. urartu* (Tu3) with chromosome 3B of *T. aestivum* (Ta3B)

S2.4.1 Comparisons of DNA and protein sequences between Tu3 and Ta3B

To ensure the genes on Tu3 and Ta3B comparable, we used the same gene prediction method described in Methods to re-annotate genes on Ta3B (**Supplementary Information Table 1**). The proteins of Tu3 were aligned to Ta3B and vice versa. We chose alignments with identity >50% and coverage >50%. In total, 3,103 genes (52.32%) on Tu3 are homologous to Ta3B. On the other hand, 3,542 genes (52.99%) on Ta3B were aligned to Tu3. At the conserved level of identity >90% and coverage >90%, only 1,721 genes (29.02%) on Tu3 were found to be homologous to Ta3B while 2,017 genes (30.18%) on Ta3B are homologous to Tu3 (**Extended Data Figure 8a**).

Peaks of TE insertion date frequency distributions located between one and two MYA on Tu3 and Ta3B. According to the fact that the ancestor of A and B genomes diverged about 6-7 MYA (Marcussen et al., 2014), we speculate that in *T. urartu* the recent retrotransposon burst occurred after split of the ancestor of A and B genomes. In addition, a recent burst of retrotransposons at around 0.1 MYA was found on Ta3B but not on Tu3 (**Extended Data Figure 8h**). The data suggest a recent genomic expansion caused by burst of retrotransposons occurred on Ta3B. This can well explain why the Ta3B is larger than Tu3.

Supplementary Information Table 1: Comparison of gene numbers and features of Tu3 and Ta3B

	**Tu3**	**Ta3B**[‡]	**Ta3B**[§]
Gene number	5,931	6,684	5,966
Max gene length	52,262	61,710	182,386
Min gene length	204	201	111
Gene length*	2,253/3,214	2,240/3,503	1,610/2,738
Transcript length*	1,216/1,402	1,188/1,381	924/1,105
Max CDS length	9,145	7,845	8,718
CDS length*	742/970	777/984	924/1,105
Protein length*	248/324	258/327	307/367
Exon length*	171/312	166/296	147/265
Intron length*	139/483	134/532	146/514
5' UTR length*	110/197	98/185	0
3' UTR length*	252/307	233/291	0
Exon# per transcript[†]	3.1/4.5	3.0/4.7	3.0/4.2
Transcript# per gene[†]	1.0/1.5	1.0/1.7	1.0/1.0

* The median/average length in base pair (bp). Tu, *T. urartu*; Ta, *T. aestivum.*

† The median/average in number.

‡ IGDBV1 gene sets

§ Ensembl gene sets

S2.4.2 Collinearity between Tu3 and Ta3B

A total of 247 syntenic blocks were detected. On average, each block covered 5.5 Mb chromosomal segment, containing 53 genes on Tu3 and 51 genes on Ta3B with about 9 collinear genes. The largest syntenic block covered a chromosomal segment of 64.3 Mb with 419 genes on Tu3 and 445 genes on Ta3B. Of them, 78 genes are collinear. All syntenic blocks covered 617 Mb (82.6%) and 651 Mb (84.1%) sequence of Tu3 and Ta3B, respectively (**Extended Data Figure 8b**). These are consistent to the results obtained from DNA collinearity from MUMmer.

On the basis of the genomic annotations and collinearity analysis, we studied DNA contractions and expansions between Tu3 and Ta3B. **Extended Data Figure 8c** showed a syntenic block composed of five consecutive collinear gene pairs. About 100 kb larger repetitive DNA were inserted on Ta3B compared to Tu3, leading to expansion in this Ta3B syntenic region. **Extended Data Figure 8d** showed another syntenic block composed of seven consecutive collinear gene pairs. We found 70 kb repetitive DNA contraction in the syntenic region of Ta3B compared to Tu3. **Extended Data Figure 8e** showed eight collinear

gene pairs, which were separated by non-collinear genes. Obvious DNA expansion involving six genes was found on Ta3B. Compared to Tu3 (0.9 Mb), Ta3B (1.6 Mb) expanded ~0.7 Mb within the syntenic regions. **Extended Data Figure 8f** showed five collinear gene pairs, which were separated by non-collinear genes. Thirteen genes were lost on Ta3B. Compared with Tu3 (8.3 Mb), Ta3B (1.3 Mb) contracted about 7 Mb. **Extended Data Figure 8g** showed collinear gene regions among Bd2 (0.3 Mb), Tu3 (4.4 Mb) and Ta3B (11.2 Mb). Compared to the segment of Tu3, large number of non-homologous genes and repeats was observed on the region of Ta3B, resulting in 7 Mb expansion.

S2.4.3 Identification of gene insertions and deletions on Tu3 and Ta3B

We identified 354 and 648 insertion genes and 393 and 213 deletion genes within the 176 syntenic blocks of Tu3 and Ta3B, respectively (**Supplementary Data 7**). Our data showed that more gene insertions occurred in collinear segments of Ta3B than that of Tu3. On the other hand, less gene deletions occurred in collinear segments of Ta3B than that of Tu3. All data indicate that Ta3B had undergone considerable gene expansion after divergence of A and B genomes.

S3 Analysis of *T. urartu* populations

S3.1 RNA-seq reads mapping and SNP calling

The average mapping ratio against the G1812 reference genome was 74.5%. After quality control, we identified 144,806 SNPs (minor allele frequency ≥0.05), involved in 22,841 genes. The average SNP frequency was 6.3 per gene. The SNPs were mainly distributed in chromosome arms (**Extended Data Figure 9a**).

S3.2 Population genetic analysis

Group I included 30 accessions, among which 13 were from Turkey, with the remaining from Armenia, Iraq, Lebanon or Syria. About 88% and 92% accessions of Group II and Group III were from Lebanon and Turkey, respectively. The three groups uncovered using STRUCTURE corresponded well to those based on phylogenetic clustering with respect to accession composition in each group. Based on π and θ calculations, the genetic diversity values for *T. urartu* accessions of Group I was highest with π= 4.01 x 10^{-5}, θ= 2.07 x 10^{-5}, Group III was found to be slightly reduced (π = 3.71 x 10^{-5}, θ = 1.81 x10^{-5}) and Group II, mainly from Lebanon, was the lowest with π =3.56 x 10^{-5}, θ = 1.77 x 10^{-5}. The genetic differentiation (*Fst*) between group I and group III was modest 0.10. There was an obvious genetic distinction between Group I and Group II, and the Group II and Group III, with the

Fst estimated to be 0.20 and 0.19, respectively (**Extended Data Figure 9c**). The accessions of *T. urartu* showed clear differences in the range of altitudes (from 400 m to 1650 m) at which they grew and were collected. The altitudes of the accessions of Group II were mainly from 1000 m, and of Group III mainly from 600 m (**Extended Data Figure 9b**). Thus, altitude may be an important environmental factor contributing to the genetic diversity in *T. urartu* population. Furthermore, when inoculated with the wheat powdery mildew pathogen *Blumeria graminis* f. sp. *tritici* (*Bgt*, race E09, Zhang et al., 2016), the majority of the accessions in the Group II (92.2%) exhibited resistance, whereas most of the accessions in Groups I and III (96.7% and 90.6%, respectively) were susceptible (**Extended Data Figure 9d**). The differences among the three groups prompted us to conduct genomic scans for selective sweeps using the 144,806 SNPs identified in 22,841 expressed genes (see above). Based on the top 1% π ratio, 141 ($\pi_{\text{Group I}}/\pi_{\text{Group II}} > 7.7$) and 143 ($\pi_{\text{Group III}}/\pi_{\text{Group II}} > 4.3$) candidate sweep signals were identified (**Extended Data Figure 9e**). The significant selective sweep signals corresponded to 239 high-confidence (HC) genes, 154 of which had functional annotations (**Supplementary Data 9**), and 23 of which were *T. urartu*-specific. Interestingly, we did not detect any significant enrichment among the HC genes for any particular GO or KEGG categories, suggesting that the functional processes that have been influenced by high altitude adaptation are quite broad. Among the 23 *T.urartu*-specific genes, one (TuG1812G0700005988) was predicted to encode a plant specific dehydrin with a calculated molecular mass of 12.8 kDa. The deduced protein carries three lysine-rich K-segments, and resembles the smallest dehydrin that has been found up-regulated by cold stress in wheat plants (Ohno et al., 2003). The population at highest altitude showed lowest genetic diversity and strong resistance to *Bgt*, indicating that Group II accessions were subjected to strong selection pressure and experienced complex adaption for improving their viability.

S4. References

Clavijo B J, Venturini L, Schudoma C, *et al*., 2017. An improved assembly and annotation of the allohexaploid wheat genome identifies complete families of agronomic genes and provides genomic evidence for chromosomal translocations. *Genome Res.*, **27**, 885-896.

Dong L, Huo N, Wang Y, *et al.*, 2016. Rapid evolutionary dynamics in a 2.8-Mb chromosomal region containing multiple prolamin and resistance gene families in *Aegilops tauschii*. *Plant J*. **87**, 495-506

Dong L, Zhang X, Liu D, *et al.*, 2010. New insights into the organization, recombination, expression and functional mechanism of low molecular weight glutenin subunit genes in bread wheat. *PLoS One* **5**, e13548.

International Brachypodium I, 2010. Genome sequencing and analysis of the model grass *Brachypodium distachyon*. *Nature* **463**, 763-768.

Ling HQ, Zhao S, Liu D, *et al.*, 2013. Draft genome of the wheat A-genome progenitor *Triticum urartu*. *Nature* **496**, 87-90.

Luo G, Ye L, Chen X, 2013. Research progress of Arabidopsis B3 transcription factor gene superfamily. *Chemistry of Life* **33**, 287-293.

Ma J, Stiller J, Zheng Z, *et al.*, 2015. Putative interchromosomal rearrangements in the hexaploid wheat (*Triticum aestivum* L.) genotype 'Chinese Spring' revealed by gene locations on homoeologous chromosomes. *BMC Evol. Biol.* **15**, 37.

Marcussen T, Sandve SR, Heier L, *et al.*, 2014. Ancient hybridizations among the ancestral genomes of bread wheat. *Science* **345**, 1250092.

Miftahudin, Ross K, Ma XF, *et al.*, 2004. Analysis of expressed sequence tag loci on wheat chromosome group 4. *Genetics* **168**, 651-663.

Murat F, Armero A, Pont C, et al., 2017. Reconstructing the genome of the most recent common ancestor of flowering plants. *Nat. Genet.* **49**, 490-496.

Pont C, Murat F, Guizard S, *et al.*, 2013. Wheat syntenome unveils new evidences of contrasted evolutionary plasticity between paleo- and neoduplicated subgenomes. *Plant J.* **76**, 1030-1044.

Ohno R, Takumi S, Nakamura C, 2003. Kinetics of transcript and protein accumulation of a low-molecular-weight wheat LEA D-11 dehydrin in response to low temperature. *J. Plant Physiol.* **160**, 193-200.

Salse J, Bolot S, Throude M, *et al.*, 2008. Identification and characterization of shared duplications between rice and wheat provide new insight into grass genome evolution. *Plant Cell* **20**, 11-24.

Swaminathan K, Peterson K, Jack T, 2008. The plant B3 superfamily. *Trends Plant Sci.* **13**, 647-655.

Zhang, J., *et al*., 2016. Coexpression network analysis of the genes regulated by two types of resistance responses to powdery mildew in wheat. *Sci. Rep.* **6**:23805.
