# Supplementary material for: Genome sequence of the progenitor of wheat A subgenome Triticum urartu
Source: Nature. 2018 May 9;557(7705):424–8. doi: 10.1038/s41586-018-0108-0 (PMC6784869; doi:10.1038/s41586-018-0108-0)
Supplement: Supplementary file 2 — Reporting Summary [file 41586_2018_108_MOESM2_ESM.pdf]

nature research

Corresponding author(s): Hong-Qing Ling, Chengzhi Liang, Daowen Wang, Aimin Zhang

Life Sciences Reporting Summary

Nature Research wishes to improve the reproducibility of the work that we publish. This form is intended for publication with all accepted life science papers and provides structure for consistency and transparency in reporting. Every life science submission will use this form; some list items might not apply to an individual manuscript, but all fields must be completed for clarity.

For further information on the points included in this form, see Reporting Life Sciences Research. For further information on Nature Research policies, including our data availability policy, see Authors & Referees and the Editorial Policy Checklist.

Please do not complete any field with "not applicable" or n/a. Refer to the help text for what text to use if an item is not relevant to your study. For final submission: please carefully check your responses for accuracy; you will not be able to make changes later.

Experimental design

1. Sample size

Describe how sample size was determined.

Fig. 1a: All identified intact LTR/Copia and LTR/Gypsy retrotransposons in T. urartu were sampled to present LTR retrotransposon bursts.
Fig. 1b: No sampling was performed.
Fig. 1c-l: The sizes of the sliding windows were chosen arbitrarily. Sliding window sizes were described in Fig. 1 legend.
Fig. 2a and Fig. 2b: Syntenic relationship between T. urartu and T. aestivum. Annotated and chromosome localized genes were sampled.
Fig. 2c: An evolutionary model of T. urartu genome. No sampling was performed.
Fig. 3a: Geographic distribution of the 147 Tu accessions from Fertile Crescent. The samples were selected arbitrary.
Fig. 3b: Sampling described in Fig. 3a above.
Fig. 3c: Sampling described in Fig. 3a above. The sample size of each group are indicated in figure and in figure legend.
Extended Data Fig. 1: No sampling was performed.
Extended Data Fig. 2: All GenBank available Tu BAC sequences were downloaded and used.
Extended Data Fig. 3: No sampling was performed.
Extended Data Fig. 4 : The sizes of the sliding windows were chosen arbitrarily. Sliding window sizes were described in Extended Data Fig. 4 legend.
Extended Data Fig. 5a: The five grass genomes were selected for their high-quality genome sequences and annotations. Fig. 5b: T. urartu specific genes identified by the standard OrthoMCL pipeline were selected to do the Gene Ontology analysis. Fig. 5c: B3 family genes, which were predicted by tool iTAK, were selected to assign to four B3 subfamilies.
Extended Data Fig. 6a: Syntenic relationship between T. urartu and T. aestivum/Ae. tauschii. Annotated and chromosome-localized genes were sampled. Fig.6b: Two BACs from Tt and Ta were selected because they have indels compared with Tu sequences. Fig.6c: Two largest scaffolds were selected in TGACv1 to compare with Tu sequences. Fig.6d: One of the chromosomes of TGACv1 was randomly selected.
Extended Data Fig. 7a - Fig. 7b: Annotated and chromosome-localized genes were sampled.
Extended Data Fig. 8a - Fig. 8g: Sampling described in Fig. 7 above.
Extended Data Fig. 9a: All DNA sequences and annotated genes were used. Fig.9b: All DNA sequences were used. Fig.9c-9g: Syntenic regions with large indels were randomly selected. Fig. 9h: Identified intact LTR retrotransposons on chromosome 3 of T. urartu and chromosome 3B of T. aestivum were ampled to present LTR retrotransposon bursts.
Extended Data Fig. 10a: No sampling was performed. The sample size of each group in figures 10b, 10d and 10g was added in figures and in the figure legends.

2. Data exclusions

Describe any data exclusions.

Methods, Lines 95-105: Reads with quality and contamination problems were excluded.
Methods, Lines 126-132: Reads with quality and contamination problems were excluded.
Methods, Lines 133-141: Reads cannot be de-convoluted were excluded.
Methods, Lines 207-223: Some scaffolds cannot be anchored on the pseudomolecules because they are lack of SNPs.
Methods, Lines 327-334: We filtered out genes which were too short or lack of evidence.
Methods, Lines 335-337: We defined high/low confidence genes.
Methods, Lines 380-391: Low quality Illumina paired-end reads were excluded.
Methods, Lines 422-425: Genes with lower sequence similarity (BLASTP E-value >= 1e-5) were excluded.
Methods, lines 477-482: Excluded SNPs with lower MAF (<0.05).
Extended Data Fig. 10e, Excluded weak candidate sweep signals.

3. Replication

Describe the measures taken to verify the reproducibility of the experimental findings.

In study on gene expression profiling of three tissues of T. urartu, we produced RNA-seq of three biological replicates for spike, two for root and four for leaf.

4. Randomization

Describe how samples/organisms/participants were allocated into experimental groups.

not involved in this work.

5. Blinding

Describe whether the investigators were blinded to group allocation during data collection and/or analysis.

not involved in this work

Note: all in vivo studies must report how sample size was determined and whether blinding and randomization were used.

6. Statistical parameters

For all figures and tables that use statistical methods, confirm that the following items are present in relevant figure legends (or in the Methods section if additional space is needed).

n/a	Confirmed	
☐	☒	The exact sample size (*n*) for each experimental group/condition, given as a discrete number and unit of measurement (animals, litters, cultures, etc.)
☒	☐	A description of how samples were collected, noting whether measurements were taken from distinct samples or whether the same sample was measured repeatedly
☒	☐	A statement indicating how many times each experiment was replicated
☐	☒	The statistical test(s) used and whether they are one- or two-sided<br>*Only common tests should be described solely by name; describe more complex techniques in the Methods section.*
☐	☒	A description of any assumptions or corrections, such as an adjustment for multiple comparisons
☐	☒	Test values indicating whether an effect is present<br>*Provide confidence intervals or give results of significance tests (e.g. P values) as exact values whenever appropriate and with effect sizes noted.*
☐	☒	A clear description of statistics including central tendency (e.g. median, mean) and variation (e.g. standard deviation, interquartile range)
☐	☒	Clearly defined error bars in all relevant figure captions (with explicit mention of central tendency and variation)

See the web collection on statistics for biologists for further resources and guidance.

▸ Software

Policy information about availability of computer code

7. Software

Describe the software used to analyze the data in this study.

All software used was described in Methods.
Customized software were deposited in
https://github.com/bma-genetics/Tu_genome_project

BWA : version 0.7.17
FPC V9.4
SMRT analysis software (v2.3.1)
MaSuRCA v2.3.2 for BAC assembly
MaSuRCA v3.2.2 for missing region assembly
BLAT v. 35x1
BLASR v4.0
IrysView v2.5.1
autonoise : IrysSolve Package v5134
RefAligner : IrysSolve Package v5134
SV detect : IrysSolve Package v5134
longranger v2.1.2
SSPACE v3.0
PBJelly v15.8.24
GATK v2.7-2
joinMAP v4.1
MSTmap v1.0
NUCmer: MUMmer Package v3.23
BLASTP : ncbi-BLAST v2.2.28
BLASTN : ncbi-BLAST v2.2.28
WU-BLASTX v3.0
Cufflinks v2.2.1
LTR_FINDER v1.0.2
RepeatModeler (v1.0.3)

RepeatMasker (v3.2.9)
Tandem Repeats Finder (TRF, v4.04)
LTRharvest (genometools, v1.5.6)
ClustalW (v2.1)
PAML (v4.8)
MISA (version 1.0)
tRNAscan-SE (version 1.23)
infernal (version 1.0)
Gramene pipeline (version 1.0)
SOAP-trans v1.03
InterProScan v5.27
OrthoMCL v2.0.9
Trimmomatic v0.35
Bowtie 2 v2.2.6
RSEM (v1.2.25)
edgeR (v3.6)
Trinity (v2.1.0)
Trinotate (v2.0.2)
SOAP.COVERAGE v2.7.7
MCScanX v1.0
ngsShoRT v2.2
TopHat2 v2.0.10
SAMtools v0.1.20
MEGA version 5
STRUCTURE v2.3.4
PopGen v1.32

For manuscripts utilizing custom algorithms or software that are central to the paper but not yet described in the published literature, software must be made available to editors and reviewers upon request. We strongly encourage code deposition in a community repository (e.g. GitHub). *Nature Methods* guidance for providing algorithms and software for publication provides further information on this topic.

Materials and reagents

Policy information about availability of materials

8. Materials availability

Indicate whether there are restrictions on availability of unique materials or if these materials are only available for distribution by a third party.

No restrictions

9. Antibodies

Describe the antibodies used and how they were validated for use in the system under study (i.e. assay and species).

No antibody was used in this study.

10. Eukaryotic cell lines

a. State the source of each eukaryotic cell line used.

No eukaryotic cell line was used in this study.

b. Describe the method of cell line authentication used.

No eukaryotic cell line was used in this study.

c. Report whether the cell lines were tested for mycoplasma contamination.

No eukaryotic cell line was used in this study.

d. If any of the cell lines used are listed in the database of commonly misidentified cell lines maintained by ICLAC, provide a scientific rationale for their use.

No commonly misidentified cell lines were used.

Animals and human research participants

Policy information about studies involving animals; when reporting animal research, follow the ARRIVE guidelines

11. Description of research animals

Provide all relevant details on animals and/or animal-derived materials used in the study.

No research animal was used in this study.

Policy information about studies involving human research participants

12. Description of human research participants

Describe the covariate-relevant population characteristics of the human research participants.

No human research participants was relevant to this study.
